# m6A methylation regulates hypoxia-induced pancreatic cancer glycolytic metabolism through ALKBH5-HDAC4-HIF1α positive feedback loop

**DOI:** 10.1038/s41388-023-02704-8

**Published:** 2023-05-06

**Authors:** Xiaoyan Liu, Maoxiao Feng, Xiaodong Hao, Zihan Gao, Zhaoxin Wu, Yuli Wang, Lutao Du, Chuanxin Wang

**Affiliations:** 1 Department of Clinical Laboratory, The Second Hospital of Shandong University, Cheeloo College of Medicine, Shandong University, 247 Beiyuan Street, Jinan, Shandong 250033 China; 2 School of Preclinical Medicine, Nanjing University of Chinese Medicine, 138 Xianlin Road, Nanjing, Jiangsu 210023 China; 3 School of pharmacy, Qingdao University, 308 Ningxia Road, Qingdao, shandong, 266071 China

**Keywords:** Cancer metabolism, Oncogenes

## Abstract

Pancreatic cancer (PC) is the most hypoxic cancer type among solid tumors. The dynamic changes of RNA N6-methyl-adenosine (m6A) contribute to tumor cells adaption to hypoxic microenvironmental. However, the regulatory mechanisms of hypoxia response in PC remains elusive. Here, we reported that the m6A demethylase ALKBH5 mediated a decrease of total mRNA m6A modification during hypoxia. Subsequently, methylated RNA immunoprecipitation sequencing (MeRIP-seq) combined with RNA sequencing (RNA-seq) revealed transcriptome-wide gene expression alteration and identified histone deacetylase type 4 (HDAC4) as a key target gene of m6A modification under hypoxic conditionds. Mechanistically, m6A methylation recognized by m6A reader-YTHDF2 enhanced the stability *of HDAC4*, and then promoted glycolytic metabolism and migration of PC cells. Our assays also demonstrated that hypoxia-induced HDAC4 enhanced HIF1a protein stability, and overexpressed HIF1a promoted transcription of ALKBH5 in hypoxic pancreatic cancer cells. Together, these results found a ALKBH5/HDAC4/HIF1α positive feedback loop for cellular response to hypoxia in pancreatic cancer. Our studies uncover the crosstalk between histone acetylation and RNA methylation modification on layer of epigenetic regulation.

## Introduction

Pancreatic cancer (PC) is the malignant tumor with the highest mortality rate among gastrointestinal tumors. It is characterized by insignificant initial symptoms, rapid deterioration, low surgical resection rate, and poor overall prognosis [1, 2]. At present, chemotherapy followed by surgical resection is a curative treatment for PC [3]. However, most patients are already at an advanced stage at the time of diagnosis, and only 15–25% patients are suitable for surgical resection [4]. Due to early metastasis and resistance to multiple chemotherapeutics, the 5-year survival rate of PC patients is only 8% even after receiving surgical resection [5]. Malignant metastasis of cancer cells is an early process in the development of pancreatic cancer and an independent factor leading to poor prognosis [6]. Therefore, it is essential to explore the genetic or epigenetic factors that lead to the development of pancreatic cancer and discover additional therapeutic targets and biomarkers to improve patient survival.

Compared with most solid tumors, PC is recognized as lowest level of oxygen [7]. The rapid proliferation of tumor cells causes an imbalance of oxygen consumption and oxygen supply in cells, resulting in an oxygen content of less than 2% in the tumor area, which forms a hypoxic microenvironment [8]. Under hypoxia, glycolytic switch occurs and tumor cells exhibit a more aggressive phenotype to cope with hypoxic environment [9–11]. Hypoxia-inducible factor-1α (HIF1α) is critical in hypoxic adaptation by promoting the transcription of glycolytic regulatory genes in cancer cells [12]. However, the role of the hypoxic microenvironment in PC development is complicated and requires further investigation.

A recent study showed that hypoxia resulted in m6A epigenetic remodeling in tumor cells [13, 14]. Conversely, the dynamic changes of RNA N6-methyl-adenosine (m6A) modification during cancer progression contribute to quick adaption to microenvironmental changes [15]. In mammalian cells, m6A modification is reversible regulated by m6A methyltransferases (METTL3, METTL14, WTAP and RBM15) and m6A demethylases (FTO and ALKBH5) [16, 17]. Additionally, the m6A modification site of mRNA can be recognized by specific RNA-binding proteins (YTHDF1/2/3, IGF2BP1/2/3, etc.), which then influence RNA function, thus conferring specific phenotypic outcomes [18, 19]. As the most abundant RNA modification in eukaryotes, m6A modification affects gene expression regulation and disease progression through regulation of RNA stability, nuclear localization, mRNA shearing, miRNA processing, and mRNA translation [20, 21]. m6A modification reveals a new disease mechanism, however, little is known about the biological significance of m6A modification under hypoxia and its underlying regulatory mechanisms in pancreatic cancer.

In the present study, we revealed a significant decrease of m6A levels in pancreatic cancer cells under hypoxia. Decreased m6A methylation promoted glycolytic metabolism and metastasis depended on ALKBH5 in hypoxic PC cells. Mechanistically, m6A methylation was recognized by YTH N6-methyladenosine RNA binding protein 2 (YTHDF2), and then enhanced the mRNA stability of histone deacetylase type 4 (HDAC4), a histone deacetylase belonging to class II of the histone deacetylase family. Interestingly, recent studies have found that HDAC4 regulates the lysine acetylation of HIF1α protein and enhances HIF1α stability in cervical cancer [22]. ALKBH5 is a nuclear 2-oxoglutarate dependent oxygenase and is a direct target of HIF1a [23]. In this study, we demonstrated that hypoxia-induced HDAC4 enhanced HIF1a protein stability through post-translational deacetylation. In addition, overexpressing HIF1a promoted the transcription of ALKBH5 in hypoxic pancreatic cancer cells. Therefore, ALKBH5, HDAC4 and HIF1α form a positive feedback loop in PC cells under hypoxic conditions and contribute to pancreatic cancer progression.

## Results

### m6A methylation was decreased in hypoxic pancreatic cancer cells

**Fig. 1:**
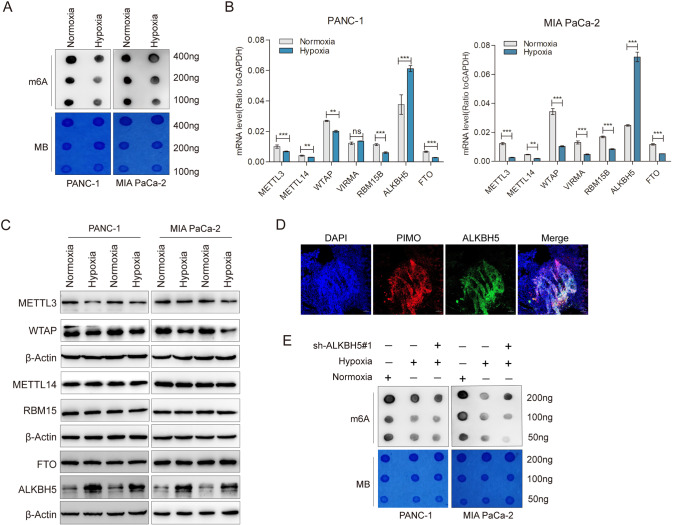
Hypoxia decreased m6A modification depended on ALKBH5 in pancreatic cancer. **A** PANC-1 and MIA PaCa-2 cells were exposed to either 20% or 1% O_2_ for 48 h. mRNA was extracted and m6A levels were determined by dot blot. RNAs were serially diluted and loaded equally with the amount of 400 ng, 200 ng and 100 ng. The intensity of dot immunoblotting (up) represented the level of m6A modification. MB, methylene blue staining (down) as loading control. **B** qRT-PCR assays were performed to analyze the mRNA levels of METTL3, WTAP, METTL14, RBM15, FTO and ALKBH5 in indicated PANC-1 and MIA PaCa-2 cells grown for 48 h under normoxia or hypoxia. **C** PANC-1 and MIA PaCa-2 cells were exposed to either 20% or 1% O_2_ for 48 h. Immunoblots showing the protein levels of METTL3, WTAP, METTL14, RBM15, FTO and ALKBH5. *n* = 3 independent experiments. **D** Immunofluorescence staining of ALKBH5 and pimonidazole (PIMO) in PANC02-derived mouse tumors. Hypoxic tumor areas were marked by PIMO staining. **E** Dot blot assays were used to measure m6A levels in sh-NC and shALKBH5 PC cells exposed to either 20% or 1% O_2_ for 48 h. Error bars indicate means ± SEM, *n* = 3, ^*^P < 0.05, ^**^P < 0.01, ^***^P < 0.001 and ns not significant.

To investigate the effects of hypoxia on m6A methylation in pancreatic cancer, we first examined the global m6A level in hypoxic cells relative to normoxic cells. A significant decrease in m6A modification of cellular mRNAs was observed under hypoxic conditions (Fig. 1A and Supplementary Fig. 1A, B). As m6A modification is installed by methyltransferases called “writer” and erased by demethylases called “eraser”, we further measured the expression levels of those m6A methylation-related enzymes in hypoxic PC cells. Notably, the transcriptional and protein levels of m6A “eraser”, ALKBH5, was obviously increased in both PANC-1 and MIA PaCa-2 cells which were exposed to hypoxia (48 h), while the opposite trend was observed for m6A “writers” including METTL3 and WTAP (Fig. 1B, C). Other main “writer” RBM15, METTL14 and “eraser” FTO were not affected. Moreover, based on the statistical analysis of transcript levels in Fig B, we found that ALKBH5 was the highest m6A modification regulatory gene under hypoxia conditions. Consistent with the in vitro findings, hypoxic areas of tumor tissue showed relatively high ALKBH5 expression in the xenograft mouse model (Fig. 1D). We then established the ALKBH5 knockdown cell lines via adenovirus infection in PANC-1 and MIA PaCa-2 cells (Supplementary Fig. 1C, D). m6A dot blot assay also confirmed that the total m6A level of mRNA was increased after interfering the expression of ALKBH5 in PANC-1 and MIA PaCa-2 cells (Supplementary Fig. 1E). We further cultured PANC-1 and MIA PaCa-2 cell lines in 1% oxygen for 48 h, m6A dot blot analysis showed that the total m6A level of mRNA was partially rescued in ALKBH5 knockdown cell lines (Fig. 1E). These results suggested that m6A modification was decreased under hypoxia depending on ALKBH5 in pancreatic cancer.

### m6A modification regulates transcriptome gene expression under hypoxic conditions

**Fig. 2:**
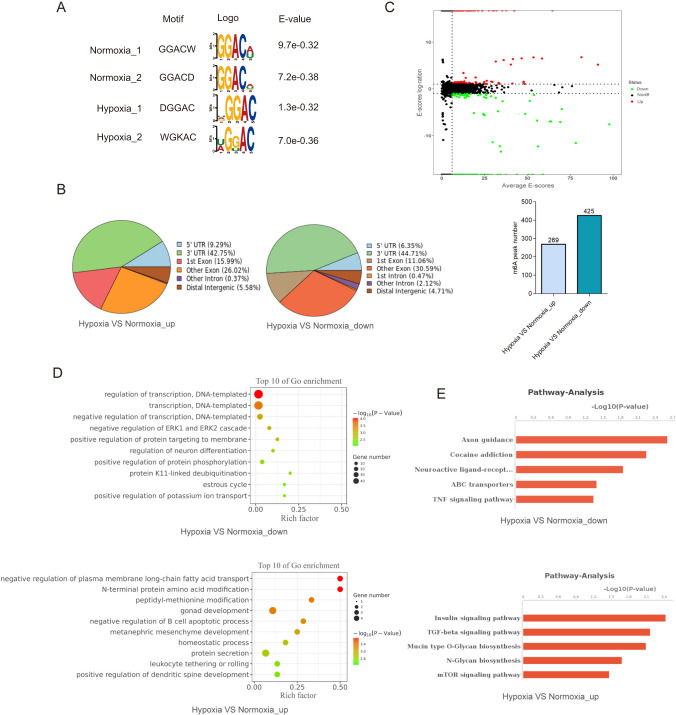
Transcriptome-wide m6A landscape under hypoxic conditions. **A** The top enriched motifs within m6A peaks in PANC-1 cells under either normoxic (20% O_2_) or hypoxic (1% O_2_) conditions. **B** Pie charts showing the m6A peak distribution in different RNA regions (CDS, 5′UTR, 3′UTR and stop codon) in PANC-1 cells under normoxia or hypoxia. **C** MAplot showing the numbers of m6A peaks under hypoxic (1% O_2_) or normoxic (20% O_2_) conditions. **D** GO enrichment map of differentially expressed m6A-containing genes between hypoxic and normoxic PANC-1 cells. **E** KEGG pathway analysis of m6A-hypo or m6A-hyper genes in response to hypoxia (1% O_2_).

To explore the regulatory role of m6A modification in gene expression under hypoxic microenvironment, we performed MeRIP-seq assay to map the m6A sites in PANC-1 cells cultured under 20% or 1% oxygen for 48 h. An average of 9825 and 8921 m6A peaks were identified from normoxic and hypoxic cells (Supplementary Fig. 2A). The m6A peaks with RRACH (R = A/G) motif were mainly enriched in the 3′ UTR region near the stop codon (Fig. 2A and Supplementary Fig. 2B). Although the motif and the pattern of m6A distribution were observed similarly in both cells (Fig. 2B), we found that there were 269 peaks differentially up-regulated and 425 peaks differentially down-regulated between groups in normoxia and hypoxia treatment (Fig. 2C). We then identified the genes covered by m6A peaks using R software package ChIPseeker. In these genes, terms of gene transcription were significantly enriched (Fig. 2D and Supplementary Fig. 2C). Moreover, genes involved in TNF and TGF-β signaling pathway were significantly enriched (Fig. 2E and Supplementary Fig. 2D). These results suggested that m6A modification may regulate gene transcription in response to hypoxic microenvironment.

**Fig. 3:**
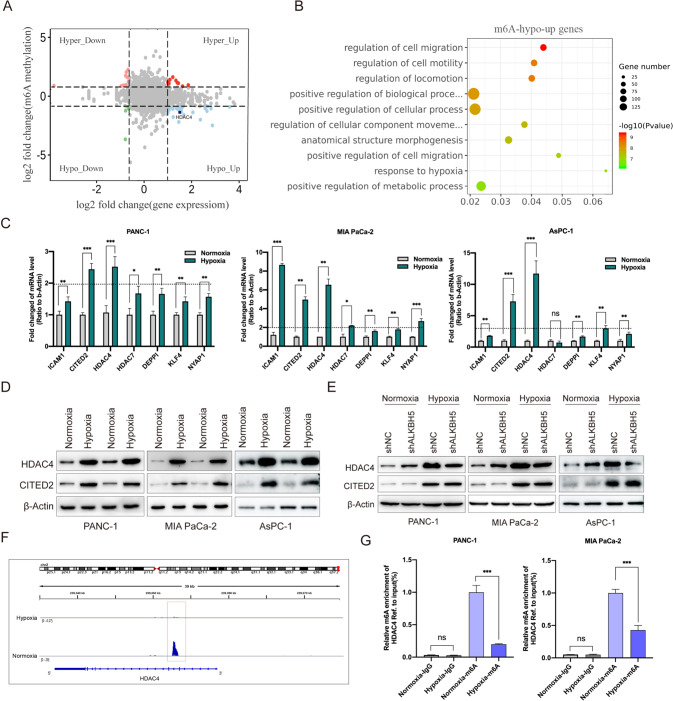
HDAC4 was identified as the candidate target of m6A under hypoxic conditions. **A** The starplot showed the distribution of genes with both differential (up or down) expression (X axis; fold change > 2 or < 0.5, *P* < 0.05) in hypoxic group compared with normoxic group. The blue dots represented up-regulated transcripts with the decreased abundance of m6A under hypoxic conditions, which were selected for the further analysis. **B** KEGG pathway analysis of m6A-hypo-up genes in response to hypoxia. **C** Real-time PCR showing the mRNA expression level of candidate target genes in PANC-1 and MIA PaCa-2 cells cultured under normoxia (20% O_2_) or hypoxia (1% O_2_) for 48 h. Error bars indicate means ± SEM, *n* = 3, ^*^P < 0.05, ^**^P < 0.01, ^***^P < 0.001 and ns not significant. **D** CITED2 and HDAC4 protein levels were measured in PANC-1 and MIA PaCa-2 cells under normoxia or hypoxia for 48 h by western blot. *n* = 3 independent experiments. **E** PANC-1 or MIA PaCa-2 cells were transfected with a shNC or sh-ALKBH5, and incubated under normoxia or hypoxia for 48 h. HDAC4 protein expression level were examined by western blot, with β-actin as the loading control. *n* = 3 independent experiments. **F** Integrative Genomics Viewer (IGV) plots showing methylation levels of representative genes upon oxygen deprivation (1% O_2_, 48 h) (gray indicates input data, blue indicates IP data). **G** Reduction in m6A modification in specific regions of *HDAC4* under hypoxic (1% O_2_) conditions, as assessed by gene-specific m6A-RIP-qPCR assays in PANC-1 and MIA PaCa-2 cells. The value obtained for the control group was set to 1. Error bars indicate means ± SEM, *n* = 3, ^*^P < 0.05, ^**^P < 0.01, ^***^P < 0.001 and ns not significant.

To systematically examine the gene expression changes resulting from the changed m6A modification under hypoxia, we combined MeRIP-seq and RNA-seq to compare the transcriptome between normoxic and hypoxic cells. Among m6A-hyper genes, 9 had reduced mRNA levels (called m6A-hyper-down genes) and 8 had increased mRNA levels (called m6A-hyper-up genes) (Fig. 3A-up). Among m6A-hypo genes, 5 had reduced mRNA levels (called m6A-hypo-down genes) and 21 had increased mRNA levels (called m6A-hypo-up genes) (Fig. 3A-down). Next, we reduced the threshold of fold change to include more differential genes. Remarkably, among the m6A-hypo-up genes, terms of cell invasion, hypoxic stress and cell metabolic processes were significantly enriched (Fig. 3B). These results suggested that m6A modification might be involved in hypoxia-regulated cellular metastasis and metabolism.

### Hypoxia-related m6A modification induced glycolysis and metastasis depending on ALKBH5

To further demonstrate that m6A modification was involved in the regulation of hypoxia-induced cell metastasis and metabolism, we cultured ALKBH5-interfering PC cells under hypoxic and normoxic conditions. Transwell assays indicated that hypoxia significantly enhanced the migratory ability of PC cells and knockdown ALKBH5 inhibited PC cell migration under hypoxic conditions, while the opposite trend was observed under normoxic conditions (Supplementary Fig. 3A, B). Hypoxia-induced elevated expression of key glycolysis regulatory genes, such as LDHA, PKM2, AIFM1, HK2, GLUT1, GLUT3, and knockdown ALKBH5 partly rescued the expression of those genes (LDHA, HK2, GLUT1 and GLUT3) under hypoxia (Supplementary Fig. 3C). Similarly, hypoxia increased lactate production (Supplementary Fig. 3D, E) and extracellular acidification rate (ECAR) (Supplementary Fig. 3F, G), and knockdown ALKBH5 partly rescued hypoxia induced glycolysis. While the glycolytic metabolism was not affected after interfering with ALKBH5 in the normoxia (Supplementary Fig. 3D–G). Moreover, we found that impaired ALKBH5 expression inhibited cell proliferation under hypoxic conditions, but promoted cell proliferation under normoxic conditions (Supplementary Fig. 3H). These results further demonstrated that the hypoxia induced m6A reduction was mediated by ALKBH5.

### HDAC4 is a mainly m6A-mediated target gene

Considering the critical role of ALKBH5 in hypoxia-regulated m6A modification, we further associatively analyzed transcripts of m6A-hypo-up and ALKBH5 target genes. The transcripts that belonged to m6A-hypo-up category and were also down-regulated upon ALKBH5 knockdown were selected for following investigations. Combining MeRIP-seq and RNA-seq data, we found 7 candidate target genes with most significant changes, namely DEPPI, KLF4, NYAP1, HDAC4, HDAC7, ICAM1 and CITED2. They were subject to preliminary validation by qPCR in normoxic and hypoxic cells. Intriguingly, only HDAC4 and CITED2 were consistently increased under hypoxic condition in all three PC cells (Fig. 3C). Western blot assay further confirmed the increased protein levels of HDAC4 and CITED2 in hypoxic cells (Fig. 3D). Moreover, western blot analysis showed that the protein level of HDAC4, instead of CITED2, was partially rescued in ALKBH5 knockdown cell lines (Fig. 3E). From our MeRIP-seq data, the m6A peak of HDAC4 mRNA on 3’UTR shrank significantly in hypoxia cells (Fig. 3F). Then MeRIP-qPCR assay revealed a decreased m6A level in *HDAC4* (Fig. 3G). Taken together, HDAC4 maybe the key gene involved in hypoxia-induced pancreatic cancer cell metastasis and glycolytic metabolism.

### Decreased m6A modification stabilized HDAC4 mRNA depending on YTHDF2

**Fig. 4:**
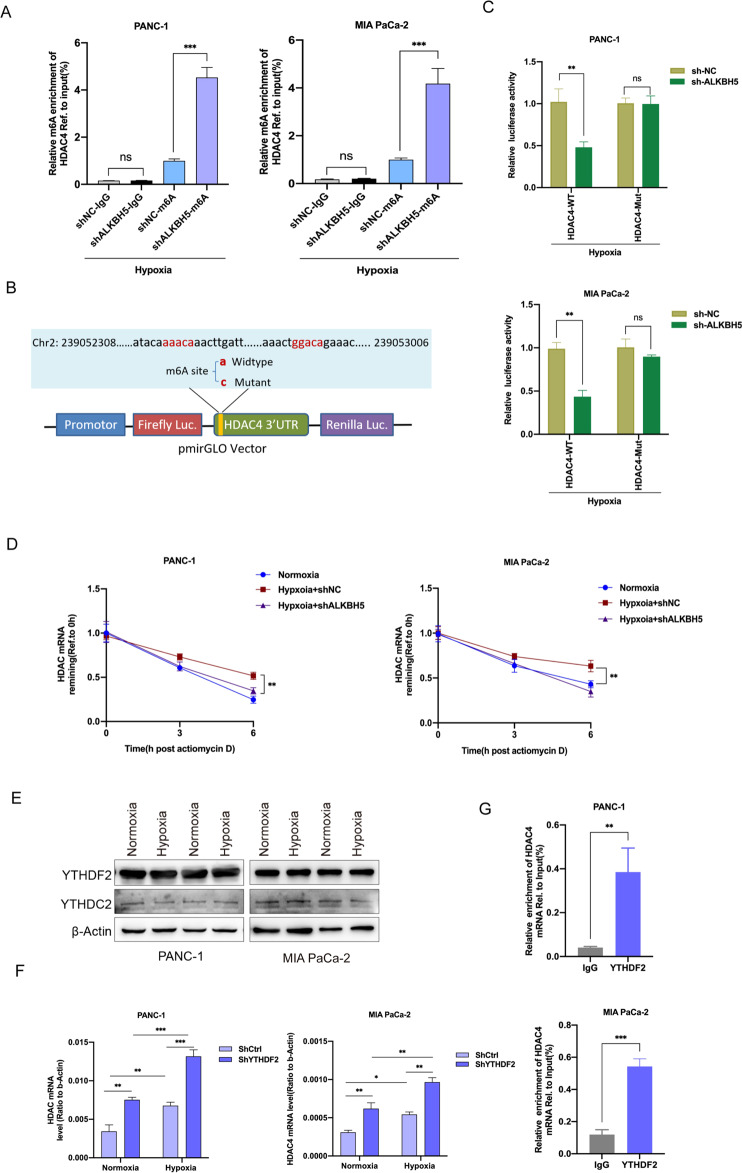
HDAC4 expression was regulated by m6A modification. **A** Reduction in m6A modification in specific regions of *HDAC4* transcripts upon ALKBH5 knockdown in PANC-1 and MIA PaCa-2 cells under hypoxia for 48 h by gene-specific m6A-RIP-qPCR assays. The value obtained for the control group was set to 1. **B** Graphical explanation for construction of luciferase reporters. The wild-type or mutant of m6A motif sequence of HDAC4 3’UTR was inserted into a pcDNA3.1 vector. **C** Dual luciferase reporter assays showing the effect of ALKBH5 on HDAC4 mRNA reporters with either wild-type or mutated m6A sites under hypoxia. **D** The stability of *HDAC4* in ALKBH5 knockdown and its corresponding control PC cells. Cells were treated with actinomycin D (2 µg/mL) at the indicated time points (0, 3 and 6 h) and were detected by qRT-PCR. Error bars are mean ± SEM. (*n* = 3). **E** Western blot showing YTHDF2 and YTHDC1 protein levels in normoxic or hypoxic PC cells. **F** The expression level of HDAC4 in YTHDF2 knockdown and its corresponding control PC cells with or without hypoxia were detected by qRT-PCR. Error bars are mean ± SEM. (*n* = 3). **G** The combination of HDAC4 and YTHDF2 was detected by RIP-qPCR analysis using anti-IgG and anti-YTHDF2 antibodies. Error bars are mean ± SEM. (*n* = 3). ^*^P < 0.05, ^**^P < 0.01, ^***^P < 0.001 and ns not significant.

Since ALKBH5 played a key role in hypoxia-regulated m6A modification, we speculated that hypoxia decreased HDAC4 mRNA m6A modification levels by ALKBH5. To substantiate this hypothesis, we conducted MeRIP-qPCR assays with specific primers aiming at potential m6A sites. The results revealed that hypoxia led to a decreased m6A levels in 3’UTR and knockdown of ALKBH5 could rescue m6A modification of HDAC4 under hypoxia (Fig. 4A). In addition, we established a dual luciferase reporter constructs with HDAC4 3′ UTR wildtype sequence (WT) or mutated m6A sites sequence (Mut) (Fig. 4B). As expected, the luciferase activity of cells transfected with wild-type 3′ UTR of HDAC4 decreased in ALKBH5-silenced cells under hypoxia, instead of the corresponding mutant fragment (Fig. 4C). Considering that the m6A modification regulated the mRNA stability, we then treated PC cells with actinomycin D to investigate whether the m6A modification affected the stability of HDAC4 mRNA. We found that hypoxia induced a slower degradation rate of HDAC4 mRNA, while the opposite effect was indicated on knockdown of ALKBH5 (Fig. 4D).

The regulatory effects of m6A modification need to be conveyed by “reader” proteins, such as YTHDFs (YTHDF1/2/3) and IGF2BPs (IGF2BP1/2/3) [24]. YTHDC1 binds to the m6A site of RNA to promote pre-mRNA splicing and mRNA nuclear export [24, 25]. YTHDC2 reduces mRNA stability while also enhances mRNA translation efficiency [24, 25]. YTHDF1 and YTHDF3 promote mRNA translation by interacting with translation initiation factors [26–28]. YTHDF2 selectively recognizes m6A modification sites of mRNA and promotes degradation of bound transcripts [26, 27, 29]. IGF2BP1, IGF2BP2, and IGF2BP3 mainly contribute to m6A-modified mRNA stabilization and translation [30]. Notedly, among these “readers”, only YTHDF2 expedites the half-life of mRNA, although, even now the functions of YTHDFs are still controversial [31]. In this study, we found the methylation level of HDAC4 mRNA was decreased under hypoxia and the expression of HDAC4 was increased. That is, m6A modification negatively regulates HDAC4 expression. According to the different functions of “reader” proteins, we speculate that YTHDF2 may be involved in the regulation of HDAC4 expression by regulating its mRNA stability. To substantiate this hypothesis, we firstly measured the expression level of YTHDC2 and YTHDF2, and the western blot assay showed, those “readers” were not affected under hypoxic conditions (Fig. 4E). Furthermore, we established YTHDF2-silenced stable cell lines (Supplementary Fig. 4A, B). Transcriptional expression level of HDAC4 were detected using a qRT-PCR assay, we found that HDAC4 mRNA expression levels were significantly increased when YTHDF2 was impaired compared with those in control cells (Fig. 4F). Thereafter, we performed RIP assays with anti-YTHDF2 antibody, as detected, HDAC4 mRNA was significantly enriched in the YTHDF2 antibody pull down sample compared with the IgG control (Fig. 4G). Together, our findings indicated that HDAC4 was governed by m6A modification depending on ALKBH5 and recognized by YTHDF2, which receded its stability under hypoxic conditions.

### HDAC4 is identified as an oncogenic driver in PC

**Fig. 5:**
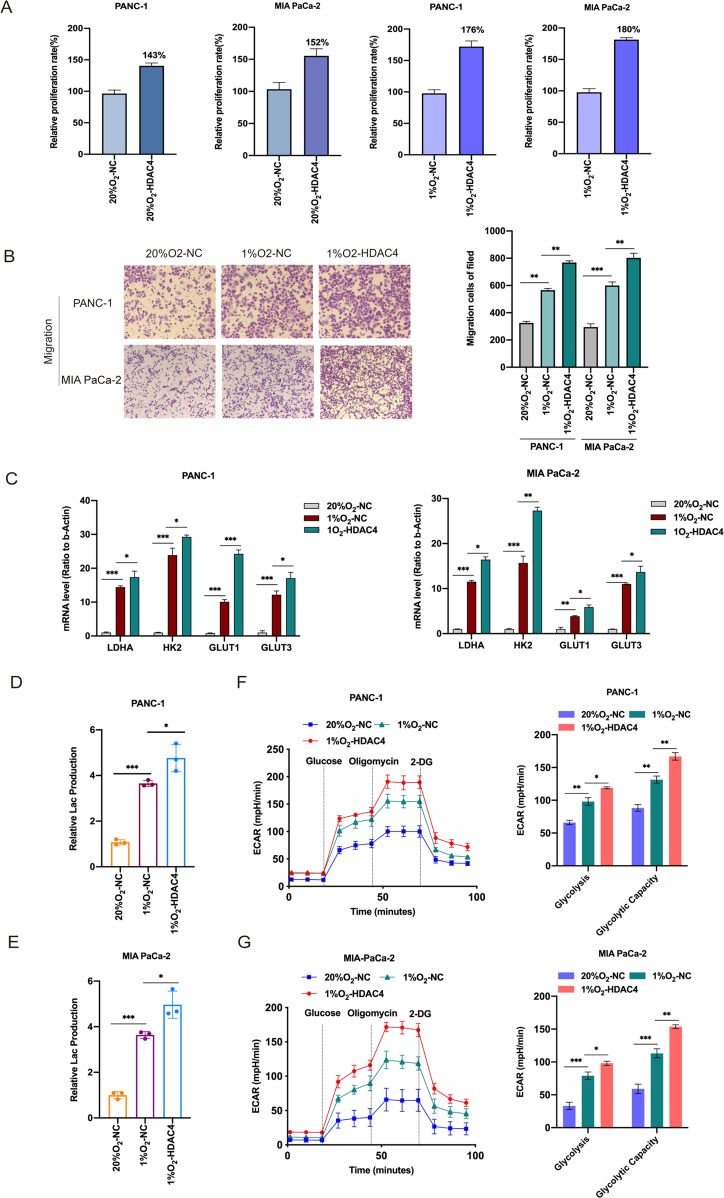
The effects of HDAC4 on PC cell metastasis and glycolysis. **A** PC cells with NC and HDAC4 overexpression were incubated under normoxia or hypoxia. CCK8 assays were used to measure cell growth, and the relative proliferation rates were calculated. **B** Transwell assays were used to investigate the changes in the migratory capability of PC cells after viral transfection with HDAC4, and cells were exposed to 20% O_2_ or 1% O_2_ for 48 h. **C** PANC-1 and MIA PaCa-2 cells with NC and HDAC4 overexpression were cultured under normoxia or hypoxia for 48 h. And the mRNA expression of glycolysis driver genes (LDHA, HK2, GLUT1 and GLUTG3) was detected by qRT-PCR assay. Error bars are mean ± SEM. (*n* = 3). **D**, **E** Lactate production was measured in HDAC4-overexpressing PANC-1 or MIA PaCa-2 cells incubated under normoxia or hypoxia. **F**, **G** The extracellular acidification rate (ECAR) assays were performed to measure the glycolytic metabolism level in HDAC4-overexpressing cells incubated under normoxia or hypoxia. The quantification of glycolysis and glycolytic capacity was summarized from raw data. The metabolic inhibitors were injected sequentially at different time points as indicated. *n* = 3-4 for each treatment group. ^*^P < 0.05, ^**^P < 0.01, ^***^P < 0.001 and ns not significant.

It has been reported that histone deacetylases (HDACs) promote tumour cell migration and the inhibition of HDACs may be a promising antitumour therapy for pancreatic cancer [32, 33]. Emerging evidence also indicates that HDAC4 mediates smoking-induced pancreatic cancer metastasis [34]. In clinical studies, elevated HDAC4 expression was significantly associated with the absence of organ metastases and tumor proliferative capacity in pancreatic adenocarcinoma [35]. These studies suggest that HDAC4 may be implicated in pancreatic malignant disease progression. In this study, we decided to systematically investigate the function of HDAC4 in pancreatic cancer under hypoxia. First, we established HDAC4-overexpression PANC-1 and MIA PaCa-2cell lines, and cultured them in 20 and 1% oxygen for 48 h (Supplementary Fig. 5A, B). We found that HDAC4 overexpression resulted in the significantly increased proliferation rate of PC cells under hypoxia compared to normoxia (Fig. 5A). The migratory assays indicated that HDAC4 overexpression significantly facilitated migratory ability of PC cells in normoxia and hypoxia (Fig. 5B, Supplementary Fig. 5C, D). Besides, HDAC4 overexpression amplified the hypoxia-induced key glycolysis regulatory genes, such as LDHA, HK2, GLUT1 and GLUT3 (Fig. 5C). Moreover, HDAC4 overexpression also increased production of lactate under normoxic and hypoxic conditions (Fig. 5D, E and Supplementary Fig. 5E). The extracellular acidification rate kinetic profiles also demonstrated the increase of glycolytic activity in HDAC4-overexpression PC cells (Fig. 5F, G and Supplementary Fig. 5F). Those data strongly suggest that HDAC4 could maintain hypoxia-induced glycolysis and metastasis in PC cells.

### Hypoxia-induced glycolysis and metastasis were reversed by silenced HDAC4 in a m6A dependent manner

**Fig. 6:**
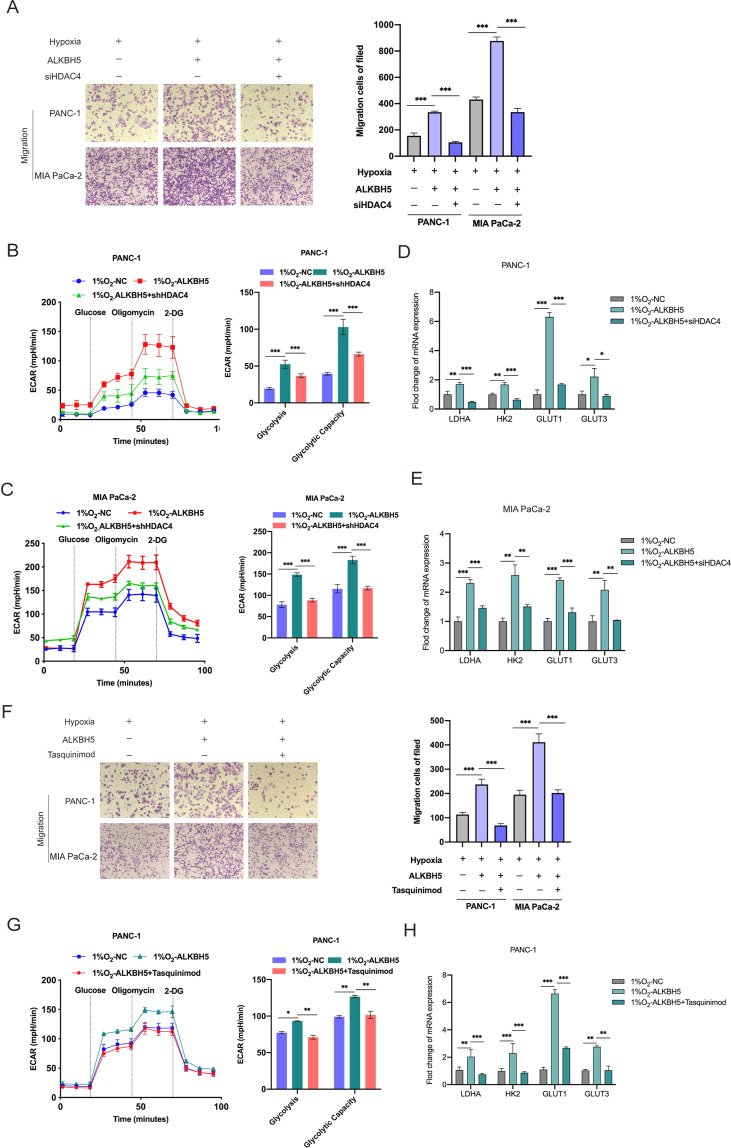
HDAC4 blockade reversed the effects of hypoxia in an m6A dependent manner. **A** ALKBH5-overexpressing PC cells transfected with the shNC or sh-HDAC4 were incubated under hypoxic conditions. Transwell assay was used to investigate the cell migration abilities. **B**, **C** ALKBH5-overexpressing PC cells transfected with the shNC or sh-HDAC4 were cultured under hypoxic conditions. The ECAR was measured to determine the glycolytic metabolism of treatment cells. Glycolytic variations (right) of glycolysis and glycolytic capacity were summarized from raw data. The metabolic inhibitors were injected sequentially at different time points as indicated. *n* = 3-4 for each treatment group. **D**, **E** ALKBH5-overexpressing PC cells transfected with the shNC or sh-HDAC4 were incubated under hypoxic conditions. The expression of a panel of glucose metabolism-related genes was detected by qRT-PCR. Error bars are mean ± SEM. (*n* = 3). **F** ALKBH5-overexpressing PANC-1 and MIA PaCa-2 cells treated with Tasquinimod under hypoxic conditions. The cell migration ability was assessed by Transwell assay. **G** ALKBH5-overexpressing PANC-1 and MIA PaCa-2 cells treated with Tasquinimod under hypoxic conditions. The glycolytic metabolism of treatment cells was measured by Seahorse XF24 system. Glycolytic variations (right) of glycolysis and glycolytic capacity were summarized from raw data. The metabolic inhibitors were injected sequentially at different time points as indicated. *n* = 3-4 for each treatment group. **H** The expression of a panel of glucose metabolism-related genes in PANC-1 and MIA PaCa-2 cells was detected by qRT-PCR. Error bars are mean ± SEM. (*n* = 3). ^*^P < 0.05, ^**^P < 0.01, ^***^P < 0.001 and ns not significant.

To confirm that the observed phenotypes were mediated by the dysregulation of ALKBH5- HDAC4 axis, we generated PC cells interfered with HDAC4 (Supplementary Fig. 6A, B). As expected, knockdown of HDAC4 significantly abolished the increased mobility ability induced by hypoxia in ALKBH5-overexpressing cells (Fig. 6A and Supplementary Fig. 6C). The Seahorse Extracellular Flux analysis showed hypoxia enhanced glycolytic capacity of ALKBH5-overexpressing cells, which could be reverted by HDAC4 silencing (Fig. 6B, C). HDAC4 silencing also abolished hypoxia-induced mRNA upregulation of key glycolysis regulatory gene in ALKBH5-overexpressing cells (Fig. 6D, E). And analogous results could be verified in ALKBH5-overexpressing cells, which were treated with tasquinimod (an inhibitor targeting HDAC4) under hypoxia (Fig. 6F–H and Supplementary Fig. 6D). These several functional rescue assays suggested that effects of hypoxia were reversed by silenced HDAC4.

### HDAC4 enhanced the stability of HIF1α in hypoxic pancreatic cancer cells

**Fig. 7:**
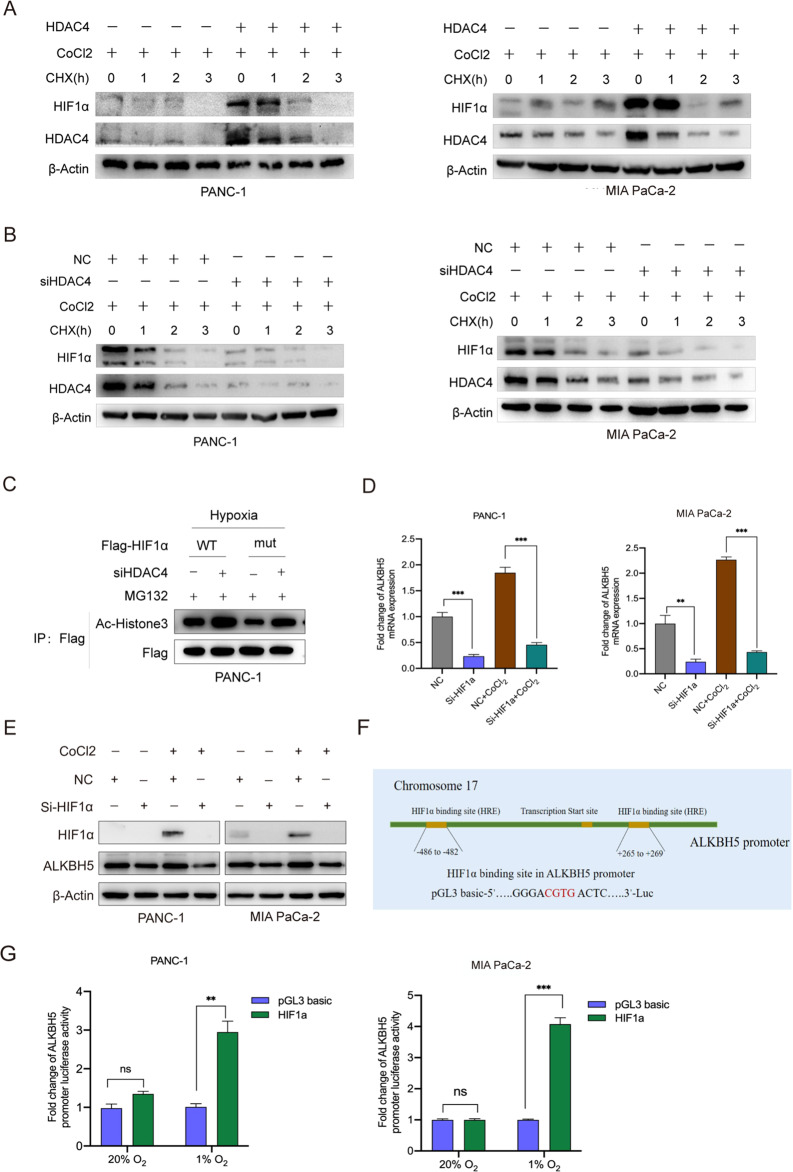
ALKBH5, HDAC4 and HIF1α regulate each other in pancreatic cancer cells. **A**, **B** PANC-1 and MIA PaCa-2 cells with HDAC4 overexpression or HDAC4 knockdown were treated with 200 µM CoCl_2_. Then, the cells were treated with 10 µg/mL cycloheximide (CHX) for the indicated time course (0, 1, 2, 3 h). The protein levels of HIF1α and HDAC4 were analyzed by western blot. **C** PC cells with or without silencing of HDAC4 expression were transfected with a wild-type Flag-HIF-1α (WT) or mutant Flag-HIF-1α (mut) plasmids for 24 h and exposed to hypoxia for 48 h. Then, the cells were treated with 5 μM MG132 for another 6 h. Cell lysates were immunoprecipitated with an anti-Flag antibody, and then immunoblotted for Flag and the acetylation form of HIF1α. **D**, **E** PC cells with HIF1α knockdown (siHIF1α) or control (NC) were treated with or without 200 µM CoCl_2_, and the expression of HDAC4 and HIF1α was detected using qRT-PCR and western blot. **F** Predicted HRE sequences in ALKBH5 promoter by JASPAR and the diagram for constructing for constructing of luciferase reporters. The wild-type or HRE mutant sequence of ALKBH5 promoter was inserted into a pcDNA3.1 vector. **G** Dual luciferase reporter assays showing the effect of HIF1α on ALKBH5 mRNA reporters with either wild-type or mutated HRE sequence under hypoxia. ^*^P < 0.05, ^**^P < 0.01, ^***^P < 0.001 and ns not significant.

HDAC4 possesses histone deacetylase activity and represses transcription when tethered to a promoter. Recent studies show that HDAC4-HIF1α axis as is an important signal pathway regulating glycolysis, apoptosis and autophagy in ovarian tumor cells during hypoxic adaptation [9, 36]. HDAC4 inhibits HIF1a ubiquitination and enhances its stability through deacetylation. Congruently, in this study, we aimed to demonstrate whether HDAC4 could enhance the stability of HIF1a in hypoxic pancreatic cancer cells. To confirm the regulatory role of HDAC4 on HIF1α, we treated HDAC4-overexpression PANC-1 and MIA PaCa-2 cell lines with Cobalt (II) chloride (CoCl_2_) to mimic hypoxia condition. Overexpression of HDAC4 significantly delayed HIF1α protein degradation (Fig. 7A). Conversely, knockdown of endogenous HDAC4 facilitated HIF1α protein degradation in hypoxic pancreatic cancer cells (Fig. 7B). We then generated a HIF1α mutant in which the lysine sites affected by HDAC4. The proteasome-specific inhibitor MG132 (10 µM) rescued the HIF1α protein from degradation in HDAC4-knockdown cells. Silenced HDAC4 caused a greater increase in acetylation of the wildtype HIF1α (HIF1α-wt) than in that of the mutant HIF1α (HIF1α-mut) (Fig. 7C). These results suggest that HDAC4 could enhance HIF1α protein stability.

### ALKBH5 was transcriptionally induced by HIF1α under hypoxic conditions

It has been reported that ALKBH5 is a nuclear 2-oxoglutarate dependent oxygenase and is a direct target of HIF1α [23, 37]. Recent study suggested that ALKBH5 promoted m6A RNA demethylation depending on HIF1α [38]. We also found that HIF1α knockdown strongly decreased the mRNA and protein levels of ALKBH5 in hypoxic pancreatic cancer cells (Fig. 7D, E). Since HIF1α binds to HRE core sequence (A/G) CGTG of target gene promoters and activates transcription, we created luciferase reporter constructs with ALKBH5 promoter (WT) or HRE sequence mutated (Mut) (Fig. 7F). The luciferase reporter assays determined HIF1α could activate ALKBH5 transcription under hypoxia (Fig. 7G). Collectively, we found that ALKBH5/HDAC4/ HIF1α form a positive feedback loop in PC cells.

### Clinical significance of the ALKBH5/HDAC4/HIF1α axis in PC

**Fig. 8:**
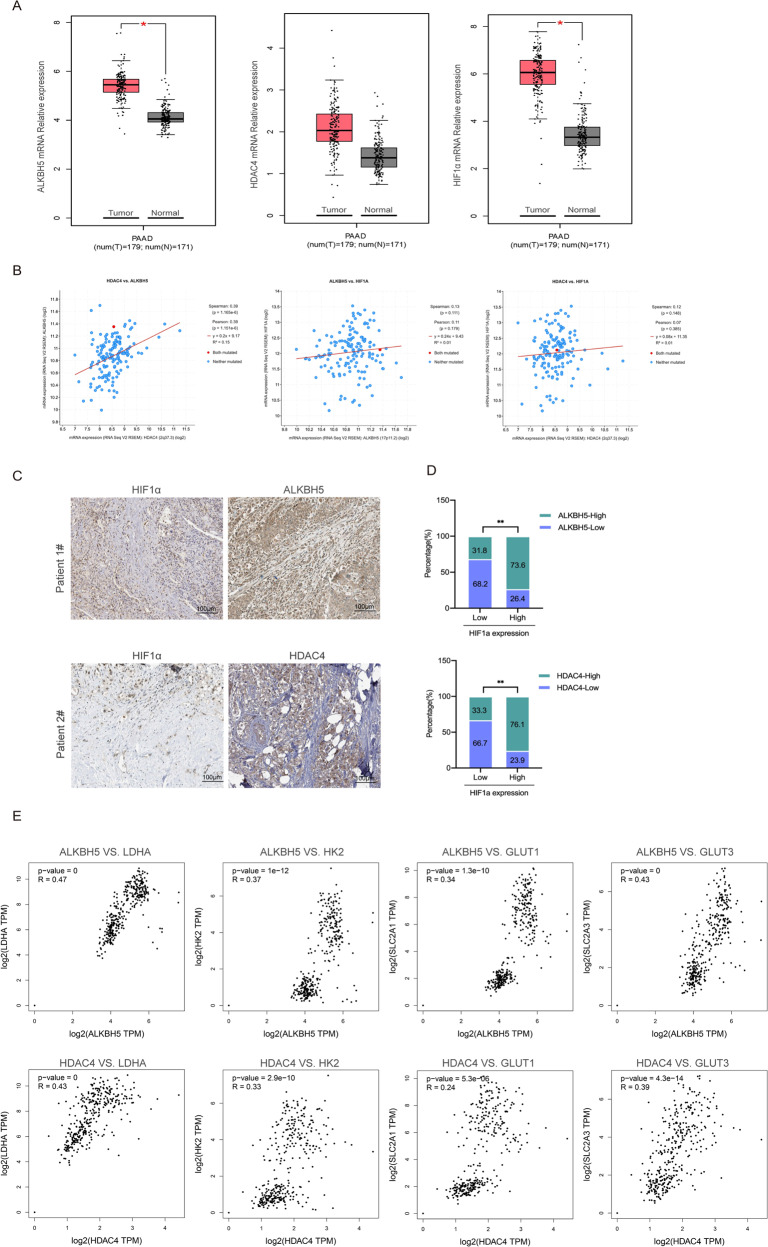
The clinical significance of the ALKBH5/HDAC4/HIF1α axis in pancreatic cancer. **A** The expression levels of ALKBH5, HDAC4 and HIF1α were analyzed in PC (*n* = 179) and normal pancreas tissues (*n* = 171) using TCGA and GTEx data. **B** The expression correlation between ALKBH5, HDAC4 and HIF1α according to TCGA PC dataset. **C** Immunohistology staining of ALKBH5, HDAC4 and HIF1α expression in 22 PC specimens. Two representative cases are shown. **D** The percentages of specimens showing low or high HIF1α expression relative to the levels of ALKBH5 and HDAC4. **E** The mRNA levels of ALKBH5 were positively correlated with hypoxia-induced marker genes GLUT1, GLUT3, HK2 or LDHA according to TCGA and GTEx dataset. Similarly, there were positive correlations between HDAC4 and hypoxia-induced marker genes. ^*^P < 0.05, ^**^P < 0.01, ^***^P < 0.001 and ns not significant.

The TCGA and GTEx datasets were was used to explore the mRNA levels of ALKBH5, HDAC4 and HIF1α in normal pancreas and PC tissues. Notably, the expression of ALKBH5 or HIF1α was increased in PC tissues, while the expression of HDAC4 was not significantly different between normal and tumor tissues (Fig. 8A). We also analyzed the correlation between the expression levels of ALKBH5, HDAC4 and HIF1α in PC tissues according to TCGA PC dataset. The result showed that ALKBH5 and HDAC4 were positively correlated in pancreatic cancer, while HIF1α was not associated with either ALKBH5 or HDAC4 on the mRNA level (Fig. 8B). Considering that ALKBH5 and HDAC4 affect the protein stability of HIF1a, we further analyzed the immunohistochemical staining of PC pathological sections according to THE HUMAN PROTEIN ATLAS data. We uncovered a significantly increased expression of ALKBH5 and HDAC4 in PC specimens (Fig. 8C). Then, we categorized the tissues specimens into HIF1α-low and HIF1α-high groups and determined their relevance in PC. Statistical analysis showed that HIF1α was positively correlated with ALKBH5 and HDAC4 on protein level (Fig. 8D). In addition, we analyzed the correlation between ALKBH5 expression and hypoxia-induced glycolytic enzymes LDHA, HK2 GLUT1 or GLUT3 using TCGA and GTEx datasets. The mRNA expression levels of ALKBH5 and hypoxia-induced glycolytic enzymes were positively correlated. Similarly, the positive correlation between HDAC4 and hypoxia-induced marker genes was also observed (Fig. 8E). These data further confirmed the relationship between ALKBH5, HDAC4 and HIF1a in PC.

## Discussion

Previous studies have found that different tumor cells have inconsistent changes in m6A modification levels in response to hypoxia. Such as, one study suggested hypoxia stimulated m6A demethylation6 in HeLa and SMMC7721 cells [31], whereas another report showed hypoxia favored m6A labelling in HCC cells [39]. The role of m6A in transcriptome reprogramming during hypoxic response in PC cells are still unclear. In this study, we systematic analyzed the alterations of m6A and its target genes in hypoxic pancreatic cancer cells by the MeRIP-seq. We found that hypoxia reduced m6A modification level of total mRNAs in a ALKBH5 dependent manner. Unexpectedly, our data suggested that ALKBH5 may act as an oncogenic driver to promote cell metastasis in hypoxia instead of serving as a tumor suppressor in normoxia. Nevertheless, the mechanism by which ALKBH5 acts inversely in normoxia and hypoxia is still unclear.

Glycolysis plays a critical role in the regulation of the tumour microenvironment, affecting biological processes such as inflammatory factor secretion, immune evasion and tumor angiogenesis [40–42]. Glycolysis is one of the primary metabolic signatures in cancers and supports the energetic requirements of sustained proliferation and metastasis [43]. Apart from DNA and histone modifications, RNA m6A modification has been recently proposed to be another important layer of epigenetic regulation in energy metabolism regulation [44, 45]. Wang et al. demonstrated that the m6A level is significantly increased due to the upregulation of methyltransferase METTL3 and then increases of glycolysis in gastric cancer [40]. Li et al. revealed that m6A could positively regulate the glycolysis of cancer cells via regulation of pyruvate dehydrogenase kinase 4 (PDK4) [46]. For PC, few studies have focused on the role of m6A modification in regulating glucose metabolism. In the present study, we found that ALKBH5 played a key role in hypoxia-regulated m6A modification. GO and GSEA analysis revealed that deletion of ALKBH5 downregulated the genes enriched in terms reflecting cellular metabolic processes. A series of in vitro experiments showed that hypoxia-induced ALKBH5 can promote glucose consumption, lactate production, and ECAR. MeRIP-seq combined with RNA-seq revealed HDAC4 as a key target gene of m6A modification under hypoxia. The decreased m6A modification of *HDAC4* 3′UTR positively regulated its mRNA stability via binding with YTHDF2. In addition, HDAC4 functioned as an oncogene to maintain hypoxia-induced proliferation, migration and glucose metabolism in pancreatic cancer cells dependent m6A modification.

**Fig. 9:**
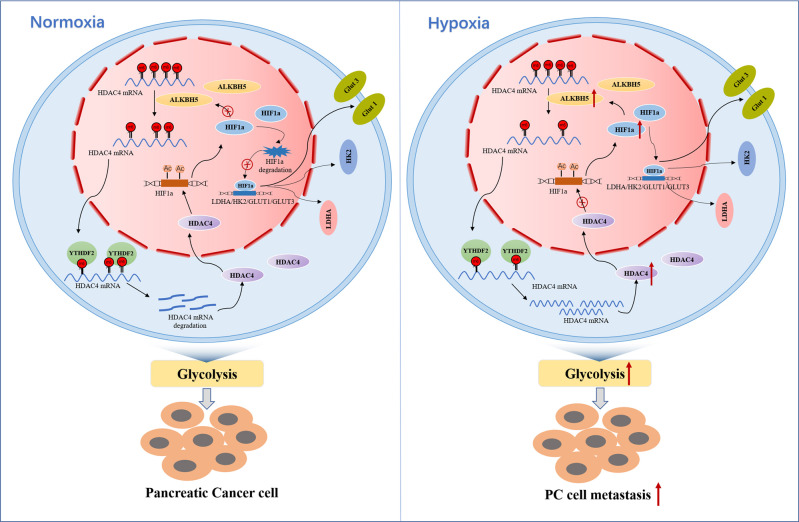
The proposed model of hypoxia-related m6A modification induced pancreatic cancer cell glycolysis and metastasis via the ALKBH5-HDAC4-HIF1α-positive feedback loop. The expression of ALKBH5 was increased under hypoxic conditions which led to increased stability of HDAC4 mRNA via m6A modification and in a YTHDF2 dependent manner. Increased HDAC4 enhanced HIF1a protein stability. And then overexpressed HIF1a promoted transcription of ALKBH5, LDHA, HK2, GLUT1 and GLUT3. Therefore, ALKBH5/HDAC4/HIF1α form a positive feedback loop, which then induces a more glycolytic metabolism and migration of PC cells under hypoxia.

Transcriptional activation mediated by HIFs is the main pathway of hypoxic adaptation [31, 47]. HIFs are composed of the stable HIF1β subunit and the oxygen-sensitive HIF1/2α subunit [48]. HIF1α is a crucial regulator of oxygen homeostasis and is degraded by the ubiquitin–proteasome pathway [49, 50]. Under hypoxic conditions, HIF1α is stabilized and binds with HIF1β to promote the transcription of downstream gene [51, 52]. Ling et al. found that the stability and transcriptional activity of HIF1α were enhanced by USP22 depended its deubiquitination activity [50]. Consistent with a previous report [9], we detected HDAC4-mediated deacetylation enhanced HIF1α stability under hypoxia. Recently, ALKBH5 was shown to be directly targeted by HIF1α and regulated by HIF2α in breast cancer cells [38]. Our study also confirmed that overexpressing HIF1α promoted ALKBH5 transcription in pancreatic cancer cells. Therefore, we demonstrated a positive feedback loop between ALKBH5, HDAC4 and HIF1α, which contributes to the maintenance of PC glycolysis under hypoxic microenvironment (Fig. 9).

Altogether, our study revealed the mechanism of m6A modification and the ALKBH5/HDAC4/HIF1α positive feedback loop in promoting glycolysis of PC. Based on our findings, the hypoxia-directed treatment maybe a novel therapeutic option for PC patients.

## Materials and methods

### RNA m6A dot blot assay

Poly(A)^+^ mRNA was enriched using GenElute messenger RNA (mRNA) Miniprep Kit (Sigma‐Aldrich). The concentration and purity of RNA were measured by an Agilent Bioanalyzer 2100. Poly(A)^+^ mRNA samples were denatured at 70 °C for 5 min and then diluted equally to 400 ng, 200 ng and 100 ng in equal volumes. After denaturation, equal volume of diluted mRNA was added into a nylon membrane (GE Healthcare). The membranes were cross-linked at 245 nm UV for three times under auto-cross-linking mode (UVP analytik jena, CL-1000M). Then, the membranes were blocked with 5% BSA for 2 h at room temperature and incubated with anti‐m6A antibody (Synaptic System, 202003) overnight at 4 °C. Membranes were washed with PBST for three times. Horseradish peroxidase-conjugated anti-rabbit immunoglobulin G (CST, 7074P2) were diluted 1:5000 and incubated with the membranes for 1 h at room temperature. Membranes were washed with PBST and the signals were detected by standard analysis of HRPO-induced chemiluminescence (ECL, Millipore). The same gradient-diluted Poly(A)^+^ mRNA was also added into the nylon membrane, stained with methylene blue (Solarbio, G1300) for 2 h, and washed with ribonuclease-free water.

### m6A sequencing and m6A-RNA immunoprecipitation assay

MeRIP sequencing was performed by Jiayin Biomedical Technology company (shanghai, China). In brief, total RNAs were isolated from PANC-1 cells exposed to hypoxia or normoxia, and then chemically fragmented into 100 ~ 200 nt. The fragmented RNA was incubated with m6A antibody (milipore) for immunoprecipitation. The enrichment of m6A containing mRNA was sent for high-throughput NGS and validated by quantitative RT–PCR. For NGS, purified RNA fragments from m6A-MeRIP were used for library construction (removing rRNA). Sequencing reads were aligned to the human genome hg38_gencode with STAR program. The m6A modification peaks were identified using software package of MetPeak. Motifs enriched with m6A peaks were identified by MEME. The R software package Guitar was used to calculate the frequency of each sample’s peak at each site in the mRNA transcript region and draw the frequency distribution map. The distribution of peaks in the functional area covered is drawn by R software package ChiIPseeker. Finally, GO and Pathway enrichment analysis of m6A peaks associated genes were carried out.

### Pimonidazole (PIMO) staining assay

2 mg pimonidazole (Hypoxyprobe) was injected intravenously into each tumor-bearing mouse and was left to circulate for 1 h before tumour resection. The obtained tumor tissue was sectioned by LEICA CM1950 (Leica). Staining of tumour sections were performed with a Hypoxyprobe RedAPC Kit (HP8-100) according to manufacturer’s instructions. Specifically, tumour sections were fixed in 4% paraformaldehyde at room temperature for 30 min. After removing fixing solution, the sections were washed 3 times with PBS and then incubated with blocking solution (goat sreum) for 1 h at room temperature. The sections were incubated with primary antibody and PIMO at 4 °C overnight. After wash, sections were incubated with corresponding secondary antibodies for 1 h at room temperature. Finally, sections were incubated with DAPI (Beyotime, China) for 5 min at room temperature, and then mounted with mounting medium containing anti-fluorescence quencher was added to mount the slides. Fluorescent images were captured using a confocal microscope (Zeiss, Germany).

### Glycolysis metabolic measurements

PC cells were seeded on Seahorse XF24 culture cell plates at a concentration of 5.0 × 10^4^ cells per well, and cells were incubated for 24 h under 20% or 1% O_2_. The probe plates were pretreated with 1 ml XF Calibrant in a non-CO_2_ incubator at 37 °C overnight. On the second day, the treatment media were removed and cultured cells were washed twice with assay medium Seahorse XF DMEM Medium (PH = 7.4) which is supplemented with 2 mM glutamine. Before detection, the culture cell plates were incubated for 1 h in a non-CO_2_ incubator at 37 °C. The final concentration of inhibitors and activators are 10 mM glucose, 1 μM Oligomycin, and 50 mM 2-DG. Cells were then assayed with an XFe24 extracellular flux analyzer (Seahorse XFe/XF). All the consumables were purchased from Agilent.

### Statistical analysis

Data were analyzed with GraphPad Prism V9.0 and SPSS V19.0. The significance of differences between groups were assessed using Student’s *t* test or χ^2^ test as appropriate. The results are presented as mean ± standard error of mean (SEM) from three independent experiments. *P* value <0.05 was considered to be statistically significant. More information of the materials and methods is in the Supplementary Materials.

## Supplementary information

Supplementary Materials

## Data Availability

All sequencing data generated in this study have been submitted to the NCBI Gene Expression Omnibus (GEO, https://www.ncbi.nlm.nih.gov/geo/) with accession number GSE218546.

## References

[1] Xia T, Wu X, Cao M, Zhang P, Shi G, Zhang J (2019). The RNA m6A methyltransferase METTL3 promotes pancreatic cancer cell proliferation and invasion. Pathol Res Pract.

[2] Li BQ, Liang ZY, Seery S, Liu QF, You L, Zhang TP (2019). WT1 associated protein promotes metastasis and chemo-resistance to gemcitabine by stabilizing Fak mRNA in pancreatic cancer. Cancer Lett.

[3] Gemenetzis G, Groot VP, Blair AB, Laheru DA, Zheng L, Narang AK (2019). Survival in Locally Advanced Pancreatic Cancer After Neoadjuvant Therapy and Surgical Resection. Ann Surg.

[4] Sung H, Ferlay J, Siegel RL, Laversanne M, Soerjomataram I, Jemal A (2021). Global Cancer Statistics 2020: GLOBOCAN Estimates of Incidence and Mortality Worldwide for 36 Cancers in 185 Countries. CA Cancer J Clin.

[5] Melchionna R, Iapicca P, Di Modugno F, Trono P, Sperduti I, Fassan M (2016). The pattern of hMENA isoforms is regulated by TGF-beta1 in pancreatic cancer and may predict patient outcome. Oncoimmunology.

[6] Ashour AA, Abdel-Aziz AA, Mansour AM, Alpay SN, Huo L, Ozpolat B (2014). Targeting elongation factor-2 kinase (eEF-2K) induces apoptosis in human pancreatic cancer cells. Apoptosis.

[7] Tan Z, Xu J, Zhang B, Shi S, Yu X, Liang C (2020). Hypoxia: a barricade to conquer the pancreatic cancer. Cell Mol Life Sci.

[8] Jing X, Yang F, Shao C, Wei K, Xie M, Shen H (2019). Role of hypoxia in cancer therapy by regulating the tumor microenvironment. Mol Cancer.

[9] Zhang Y, Ren YJ, Guo LC, Ji C, Hu J, Zhang HH (2017). Nucleus accumbens-associated protein-1 promotes glycolysis and survival of hypoxic tumor cells via the HDAC4-HIF-1alpha axis. Oncogene.

[10] Roma-Rodrigues C, Mendes R, Baptista PV, Fernandes AR (2019). Targeting Tumor Microenvironment for Cancer Therapy. Int J Mol Sci.

[11] Kong F, Kong X, Du Y, Chen Y, Deng X, Zhu J (2017). STK33 Promotes Growth and Progression of Pancreatic Cancer as a Critical Downstream Mediator of HIF1alpha. Cancer Res.

[12] Lee HJ, Jung YH, Oh JY, Choi GE, Chae CW, Kim JS (2019). BICD1 mediates HIF1alpha nuclear translocation in mesenchymal stem cells during hypoxia adaptation. Cell Death Differ.

[13] Gu C, Wang Z, Zhou N, Li G, Kou Y, Luo Y (2019). Mettl14 inhibits bladder TIC self-renewal and bladder tumorigenesis through N(6)-methyladenosine of Notch1. Mol Cancer.

[14] Li Q, Ni Y, Zhang L, Jiang R, Xu J, Yang H (2021). HIF-1alpha-induced expression of m6A reader YTHDF1 drives hypoxia-induced autophagy and malignancy of hepatocellular carcinoma by promoting ATG2A and ATG14 translation. Signal Transduct Target Ther.

[15] Dong F, Qin X, Wang B, Li Q, Hu J, Cheng X (2021). ALKBH5 Facilitates Hypoxia-Induced Paraspeckle Assembly and IL8 Secretion to Generate an Immunosuppressive Tumor Microenvironment. Cancer Res.

[16] Chen Y, Peng C, Chen J, Chen D, Yang B, He B (2019). WTAP facilitates progression of hepatocellular carcinoma via m6A-HuR-dependent epigenetic silencing of ETS1. Mol Cancer.

[17] He L, Li H, Wu A, Peng Y, Shu G, Yin G (2019). Functions of N6-methyladenosine and its role in cancer. Mol Cancer.

[18] Lobo J, Costa AL, Cantante M, Guimaraes R, Lopes P, Antunes L (2019). m(6)A RNA modification and its writer/reader VIRMA/YTHDF3 in testicular germ cell tumors: a role in seminoma phenotype maintenance. J Transl Med.

[19] Fu Y, Zhuang X (2020). m(6)A-binding YTHDF proteins promote stress granule formation. Nat Chem Biol.

[20] Taketo K, Konno M, Asai A, Koseki J, Toratani M, Satoh T (2018). The epitranscriptome m6A writer METTL3 promotes chemo- and radioresistance in pancreatic cancer cells. Int J Oncol.

[21] Oerum S, Meynier V, Catala M, Tisne C (2021). A comprehensive review of m6A/m6Am RNA methyltransferase structures. Nucleic Acids Res.

[22] Geng H, Harvey CT, Pittsenbarger J, Liu Q, Beer TM, Xue C (2011). HDAC4 protein regulates HIF1alpha protein lysine acetylation and cancer cell response to hypoxia. J Biol Chem.

[23] Thalhammer A, Bencokova Z, Poole R, Loenarz C, Adam J, O’Flaherty L (2011). Human AlkB homologue 5 is a nuclear 2-oxoglutarate dependent oxygenase and a direct target of hypoxia-inducible factor 1alpha (HIF-1alpha). PLoS One.

[24] Lan Q, Liu PY, Bell JL, Wang JY, Huttelmaier S, Zhang XD (2021). The Emerging Roles of RNA m(6)A Methylation and Demethylation as Critical Regulators of Tumorigenesis, Drug Sensitivity, and Resistance. Cancer Res.

[25] Mao Y, Dong L, Liu XM, Guo J, Ma H, Shen B (2019). m(6)A in mRNA coding regions promotes translation via the RNA helicase-containing YTHDC2. Nat Commun.

[26] Liu J, Dou X, Chen C, Chen C, Liu C, Xu MM (2020). N (6)-methyladenosine of chromosome-associated regulatory RNA regulates chromatin state and transcription. Science.

[27] Yu J, She Y, Yang L, Zhuang M, Han P, Liu J (2021). The m(6) A Readers YTHDF1 and YTHDF2 Synergistically Control Cerebellar Parallel Fiber Growth by Regulating Local Translation of the Key Wnt5a Signaling Components in Axons. Adv Sci (Weinh).

[28] Wang X, Zhao BS, Roundtree IA, Lu Z, Han D, Ma H (2015). N(6)-methyladenosine Modulates Messenger RNA Translation Efficiency. Cell.

[29] Wang X, Lu Z, Gomez A, Hon GC, Yue Y, Han D (2014). N6-methyladenosine-dependent regulation of messenger RNA stability. Nature.

[30] Huang H, Weng H, Sun W, Qin X, Shi H, Wu H (2018). Recognition of RNA N(6)-methyladenosine by IGF2BP proteins enhances mRNA stability and translation. Nat Cell Biol.

[31] Wang YJ, Yang B, Lai Q, Shi JF, Peng JY, Zhang Y (2021). Reprogramming of m(6)A epitranscriptome is crucial for shaping of transcriptome and proteome in response to hypoxia. RNA Biol.

[32] Aghdassi A, Sendler M, Guenther A, Mayerle J, Behn CO, Heidecke CD (2012). Recruitment of histone deacetylases HDAC1 and HDAC2 by the transcriptional repressor ZEB1 downregulates E-cadherin expression in pancreatic cancer. Gut.

[33] Edderkaoui M, Chheda C, Soufi B, Zayou F, Hu RW, Ramanujan VK (2018). An Inhibitor of GSK3B and HDACs Kills Pancreatic Cancer Cells and Slows Pancreatic Tumor Growth and Metastasis in Mice. Gastroenterology.

[34] Yang J, Chheda C, Lim A, Hauptschein D, Zayou L, Tang J (2022). HDAC4 Mediates Smoking-Induced Pancreatic Cancer Metastasis. Pancreas.

[35] Giaginis C, Damaskos C, Koutsounas I, Zizi-Serbetzoglou A, Tsoukalas N, Patsouris E (2015). Histone deacetylase (HDAC)-1, -2, -4 and -6 expression in human pancreatic adenocarcinoma: associations with clinicopathological parameters, tumor proliferative capacity and patients’ survival. BMC Gastroenterol.

[36] Zhang X, Qi Z, Yin H, Yang G (2019). Interaction between p53 and Ras signaling controls cisplatin resistance via HDAC4- and HIF-1alpha-mediated regulation of apoptosis and autophagy. Theranostics.

[37] Xia X, Lemieux ME, Li W, Carroll JS, Brown M, Liu XS (2009). Integrative analysis of HIF binding and transactivation reveals its role in maintaining histone methylation homeostasis. Proc Natl Acad Sci USA.

[38] Zhang C, Samanta D, Lu H, Bullen JW, Zhang H, Chen I (2016). Hypoxia induces the breast cancer stem cell phenotype by HIF-dependent and ALKBH5-mediated m(6)A-demethylation of NANOG mRNA. Proc Natl Acad Sci USA.

[39] Hou J, Zhang H, Liu J, Zhao Z, Wang J, Lu Z (2019). YTHDF2 reduction fuels inflammation and vascular abnormalization in hepatocellular carcinoma. Mol Cancer.

[40] Wang Q, Chen C, Ding Q, Zhao Y, Wang Z, Chen J (2020). METTL3-mediated m(6)A modification of HDGF mRNA promotes gastric cancer progression and has prognostic significance. Gut.

[41] Pate KT, Stringari C, Sprowl-Tanio S, Wang K, TeSlaa T, Hoverter NP (2014). Wnt signaling directs a metabolic program of glycolysis and angiogenesis in colon cancer. EMBO J.

[42] Pavlova NN, Thompson CB (2016). The Emerging Hallmarks of Cancer Metabolism. Cell Metab.

[43] Ganapathy-Kanniappan S, Geschwind JF (2013). Tumor glycolysis as a target for cancer therapy: progress and prospects. Mol Cancer.

[44] Lin Z, Niu Y, Wan A, Chen D, Liang H, Chen X (2020). RNA m(6) A methylation regulates sorafenib resistance in liver cancer through FOXO3-mediated autophagy. EMBO J.

[45] Jiang X, Liu B, Nie Z, Duan L, Xiong Q, Jin Z (2021). The role of m6A modification in the biological functions and diseases. Signal Transduct Target Ther.

[46] Li Z, Peng Y, Li J, Chen Z, Chen F, Tu J (2020). N(6)-methyladenosine regulates glycolysis of cancer cells through PDK4. Nat Commun.

[47] Semenza GL (2012). Hypoxia-inducible factors in physiology and medicine. Cell.

[48] Keith B, Johnson RS, Simon MC (2011). HIF1alpha and HIF2alpha: sibling rivalry in hypoxic tumour growth and progression. Nat Rev Cancer.

[49] Tiwari A, Tashiro K, Dixit A, Soni A, Vogel K, Hall B (2020). Loss of HIF1A From Pancreatic Cancer Cells Increases Expression of PPP1R1B and Degradation of p53 to Promote Invasion and Metastasis. Gastroenterology.

[50] Ling S, Shan Q, Zhan Q, Ye Q, Liu P, Xu S (2020). USP22 promotes hypoxia-induced hepatocellular carcinoma stemness by a HIF1alpha/USP22 positive feedback loop upon TP53 inactivation. Gut.

[51] Lisy K, Peet DJ (2008). Turn me on: regulating HIF transcriptional activity. Cell Death Differ.

[52] Stegen S, Laperre K, Eelen G, Rinaldi G, Fraisl P, Torrekens S (2019). HIF-1alpha metabolically controls collagen synthesis and modification in chondrocytes. Nature.

