# Supplementary material for: m6A methylation regulates hypoxia-induced pancreatic cancer glycolytic metabolism through ALKBH5-HDAC4-HIF1α positive feedback loop
Source: Oncogene. 2023 May 6;42(25):2047–60. doi: 10.1038/s41388-023-02704-8 (PMC10275754; doi:10.1038/s41388-023-02704-8)
Supplement: Supplementary file 1 — Supplementary Materials [file 41388_2023_2704_MOESM1_ESM.docx]

**Supplementary Information**

**Supplementary Materials and Methods**

**m6A quantification**

Total m6A mRNA levels were measured by ELISA assay using the EpiQuik m6A RNA Methylation Quantification kit (Epigentek, Farmingdale, NY, USA). Measurements were performed according to the manufacturer’s instructions. Specifically, 200 ng of purified mRNA was added for the analysis of each sample.

**Cell culture**

Human PC cell lines (PANC-1, Aspc-1 and MIA PaCa-2) were cultured in high-glucose Dulbecco’s modified Eagle’s medium (Gibco) supplemented with 10% fetal bovine serum (BI), 100 mg/mL penicillin G and 100 µg /mL streptomycin (Solarbio, China) at 37°C and 5% CO_2_. Hypoxic-treated cells were cultured in a bis-gas incubator with 1% O_2_ and 5% CO_2_, balanced with N_2_.

**Western blot**

PC cells were harvested and lysed with RIPA lysis buffer (Beyotime, China) containing the protease inhibitor cocktail (Roche, Basel, Switzerland). The protein concentration was determined using the Enhanced BCA Protein Assay Kit (Beyotime). And then proteins were denatured at 100°C for 10min. Protein were loaded on SDS-PAGE and transferred to PVDF membranes (Merck-Millipore, Germany) by an electroblot apparatus. The membranes were blocked with 5% BSA (Solarbio, China) in 1% Tween 20 phosphate-buffered saline (TBST) for 1 h at room temperature and then were incubated with primary antibodies at 4 °C overnight. Then, the membranes were subsequently washed and incubated with secondary antibodies (Cell Signaling Technology) for 1h at room temperature. Finally, the signals were detected by standard analysis of HRPO-induced chemiluminescence. Antibodies used in this study were obtained from Cell Signaling Technology (Boston, USA), Proteintech (Chicago, USA) and Abcam (Cambridge, UK). The antibodies were anti-ALKBH5 (ab195377, Abcam), anti-FTO (31687S, CST), anti-METTL3(96391, CST), anti-METTL14 (51104, CST), anti-WTAP (56501, CST), anti-RBM15(10587-1-AP, Proteintech), anti-N6-methyladenosine (m6A) (ab208577, Abcam), anti-HIF-1α (36169T CST), anti-HDAC7 (33418s, CST), β-Actin (8457, CST), HDAC4(5392T,CST), anti-mouse IgG, HRP-linked Antibody (7076S,CST) and anti-rabbit IgG, HRP-linked Antibody (7074P2,CST).

**Quantitative real-time RT-PCR**

Total RNA was isolated using Trizol reagent according to the manufacturer’s instructions (Invitrogen, Carlsbad, CA, USA). Then, the RNA was reversely transcribed using the PrimeScript RT Reagent Kit (Takara, Japan). The resulting cDNA was used as the templates for quantitative real-time PCR analysis using SYBR Premix Ex Taq (Takara) QuantStudio™ 5 Real-Time PCR System (Thermofisher QuantStudio 5). The relative RNA expression levels were analyzed by 2^-ΔΔCt^ method, with the levels normalized to β-Actin expression level. The specific primers are listed in supplementary Table 1.

**Cell proliferation assays**

CCK8 assay was used to evaluate cell proliferation ability. PC cells were seeded at 1,000 cells per well in 96-well plates, and the hypoxic-treated cells were exposed to 1% O_2_ hypoxic environment. Cell number was monitored with Cell Counting Kit-8 (CK04, Dojindo, Japan) at the 48h time points. 10 μl of CCK-8 solution was added into per well and incubated for 2h at 37°C according to the manufacturer’s instructions. The spectrophotometric absorbance at OD450 was determined by the SpectraMax i3X (Molecular Devices, USA).

**Cell migration assays**

For the Transparent PET membrane, 5×10^4^ cells were re-suspended in 200 μl serum-free media and seeded into the upper chamber of transwell with 8-μm pore size chamber inserts (353097, Corning), and 800 μl medium containing 20% FBS was added to the bottom chamber. After 24 h of normoxic or hypoxic culture, migrated cells were fixed with methanol and stained with crystal violet. The images were captured using inversion microscope (Zeiss, Germany). For the Fluoblock membrane, 8×10^4^ cells expressing green fluorescent protein were re-suspended in 200 μl serum-free media and seeded into the upper chamber of transwell with 8-μm pore size chamber inserts (351152, Corning). And then 800 μl medium containing 20% FBS was added to the bottom chamber. After 24 h of normoxic and hypoxic culture, fluorescent images were captured using a confocal microscope (Zeiss, Germany).

**Animal procedures and model**

A number of 5×10^6^ PANC02 cells were resuspended in 100 μl of PBS and subcutaneously injected into the right flank of 6-week old male C57 mice. The mice were euthanized when the tumor length reached about 1.5 cm. Obtained tumor tissue was used for PIMO staining assay.

**Constructs and transfections**

Stable knockdown of ALKBH5 or YTHDF2 in PC cell lines was generated by adenovirus-based shRNA delivery. Specific target shRNAs were subcloned into lentiviral vector pADM-U6-shRNA-mCMV-copGFP (Ji-Nan Weizhen), and a non-target shRNA was used as a negative control. The shRNA sequences employed in this study are listed in supplementary Table 2. The siRNAs targeting HDAC4 or HIF1α were transiently transfected into PC cell lines by using Lipofectamine RNAiMAX (Invitrogen) following manufacturer’s instructions. A non-target siRNA was used as negative control. The siRNA sequences are listed in supplementary Table 2.

**Luciferase reporter assay**

The 3′UTR sequence of HDAC4 was subcloned into the dual-luciferase vector pmirGLO to construct pmiRGLO-WT-3′UTR plasmids. Similarly, the m6A recognition site which is mutagenesis from A to T was subcloned into pmiGLO to construct pmiRGLO- Mut-3′UTR plasmids. PC cells were transfected with the luciferase reporter, pRL-TK Renilla luciferase construct, or ALKBH5 expression vectors in 24-well plate. After transfection for 48 h, the luciferase activities of firefly and Renilla were analyzed with the Dual-Luciferase Assay kit (Promega). The firefly luciferase activity values were normalized to the Renilla luciferase activity values to represent the expression efficiency.

**RNA immunoprecipitation (RIP) assay**

The RIP assay was performed using the Imprint RNA Immunoprecipitation Kit (Millipore, USA) according to the manufacturer’s instructions. Briefly, PC cells were lysed with RIP lysis buffer, and the remaining supernatant was incubated with magnetic beads conjugated with 5 μg anti-YTHDF2 or anti-YTHDC2 at 4 °C for 3–6 h. The anti-lgG was used as negative control. Immunoprecipitated RNA was eluted, purified, and dissolved in RNase-free water, which was further measured through qRT-PCR. The enrichment of a transcript was calculated as the ratio between its amount in IP to that in the input, yielded from same number of cells.

**RNA stability assay**

PC cells were exposed to hypoxia or normoxia, and treated with actinomycin D (MCE) at a concentration of 5 mg/ml. After incubation at the indicated times, cells were collected, and RNA was isolated for RT-qPCR. The half-life (t1/2) of HDAC4 mRNA were calculated using ln2/slope and β-Actin was used for normalization.

**Lactate production assays**

Lactate were measured using glucose assay kit and lactate assay kit (Solarbio, China), respectively. The procedures followed the manufacturer’s protocols. In brief, cells were incubated for 24 h under 20% or 1% O_2_ and then lysed with lysis buffer. The supernatant was collected for the detection of lactate accumulation. The absorbances at 450 nm were recorded using the SpectraMax i3X (Molecular Devices, USA) for lactate production.

**Supplementary Figures**

**
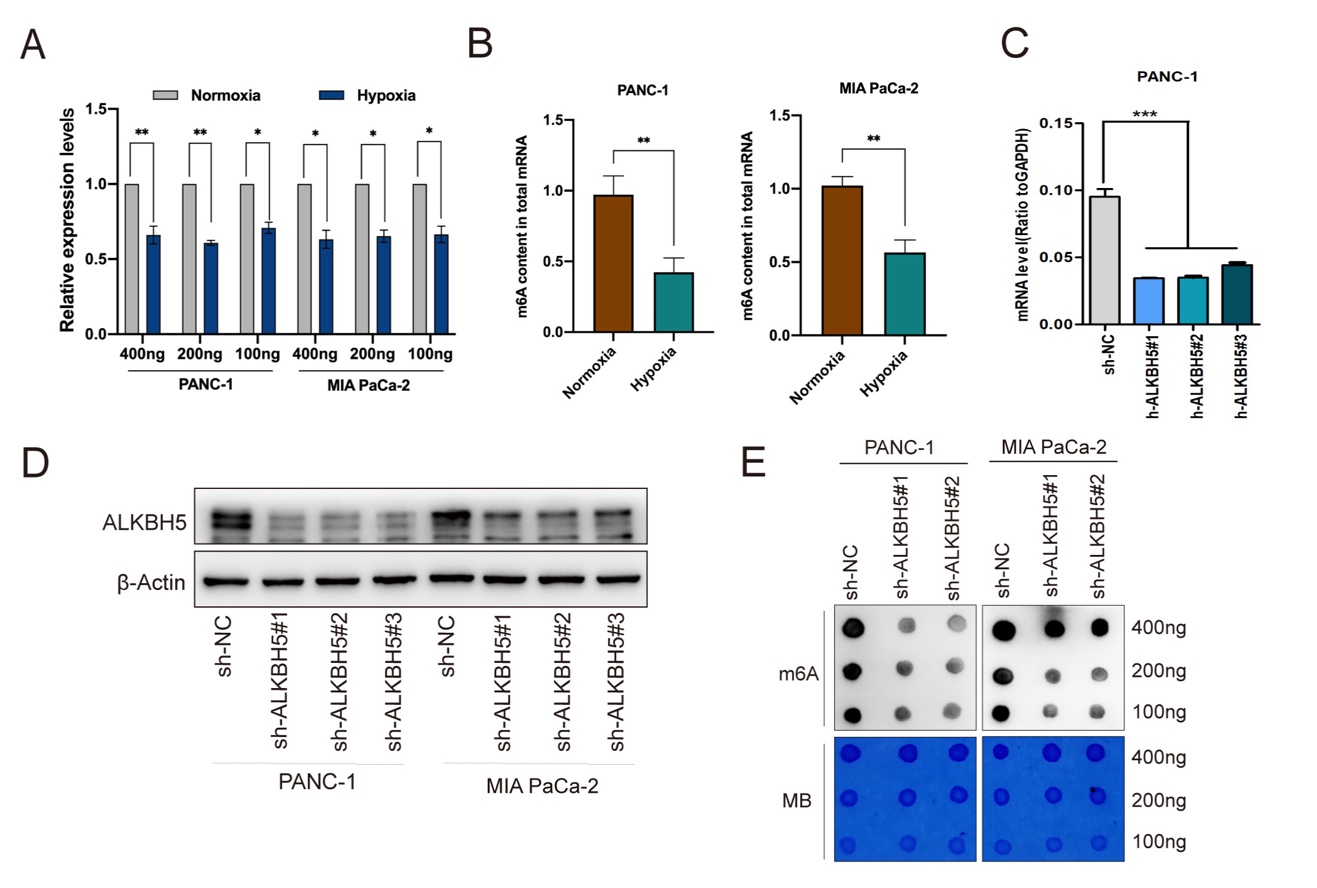
**

**Figure S1.** Hypoxia increased the expression of ALKBH5 and decreased m6A level of total mRNA. (**A**) The spot intensity of Fig1A was quantified using ImageJ software. (**B**) The m6A levels of total RNA in normoxic or hypoxic PC cells were assessed by ELISA assay with an EpiQuik m6A RNA Methylation Quantification kit. (**C**) Real-time PCR verified of ALKBH5 mRNA expression in PC cells transduced with knockdown ALKBH5. (**D**) Western blot assays were used to verify the reduced expression of ALKBH5 in PANC-1 and MIA PaCa-2 cells. (**E**) The m6A levels of mRNA in PC cells cultured under normoxia or hypoxia were normoxia or hypoxia were determined by dot blot assay. n=3 independent experiments. *P < 0.05, **P < 0.01, ***P < 0.001 and ns not significant.


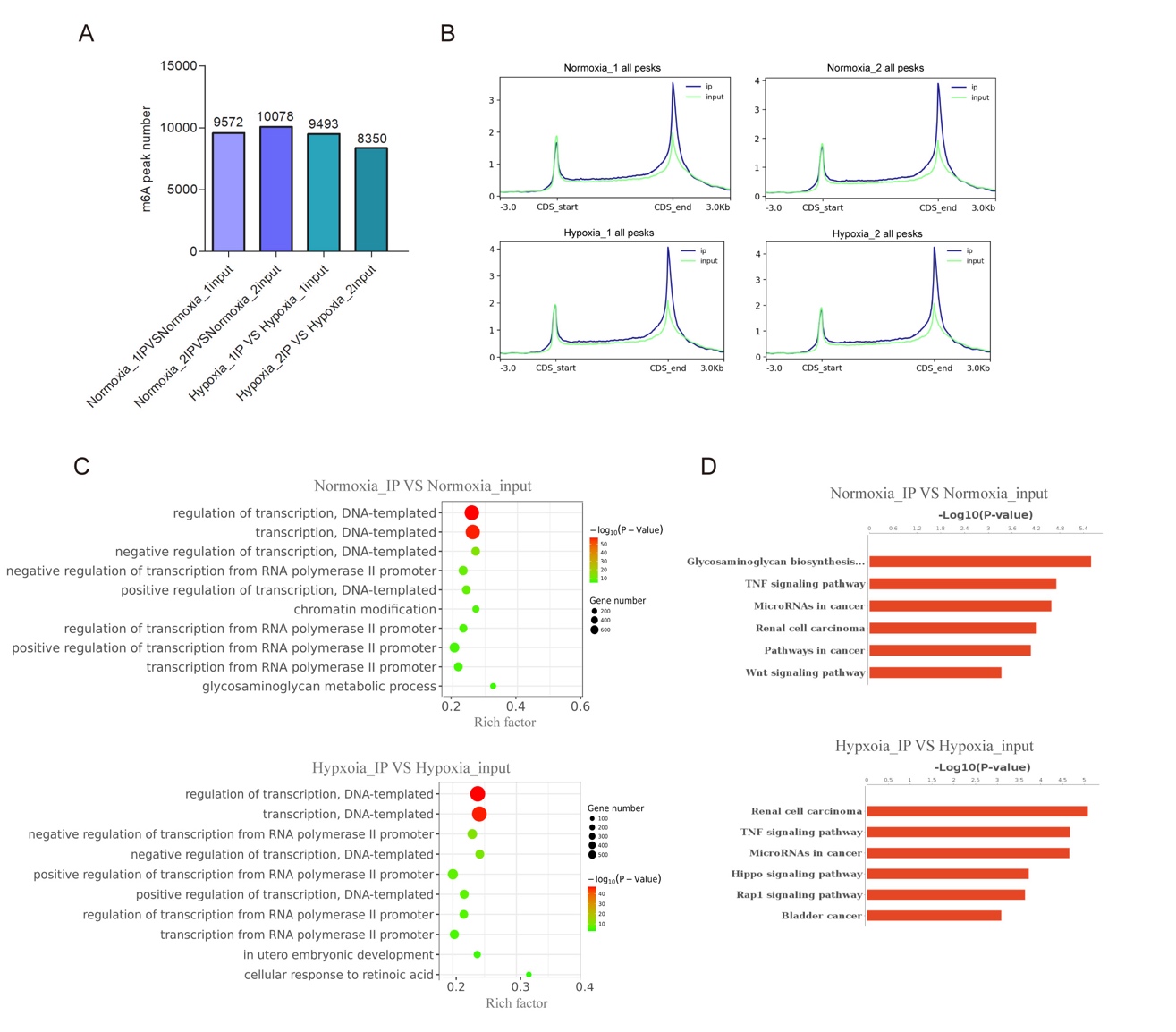


**Figure S2.** Identification of gene expression in m6A-epitranscriptome of PC cells under hypoxia. (**A**) Number of peaks of m6A in MeRIP-seq in response to hypoxia (1% O_2_). (**B**) Curve chart showing the m6A peak distribution in different RNA regions in PANC-1 cells which were cultured under normoxia or hypoxia for 48 h. (**C**) Gene ontology (GO) enrichment analysis of genes with m6A modification under normoxia (20% O_2_) or hypoxia (1% O_2_). (**D**) KEGG pathway analysis of m6A-modified genes under normoxia (20% O_2_) or hypoxia (1% O_2_).


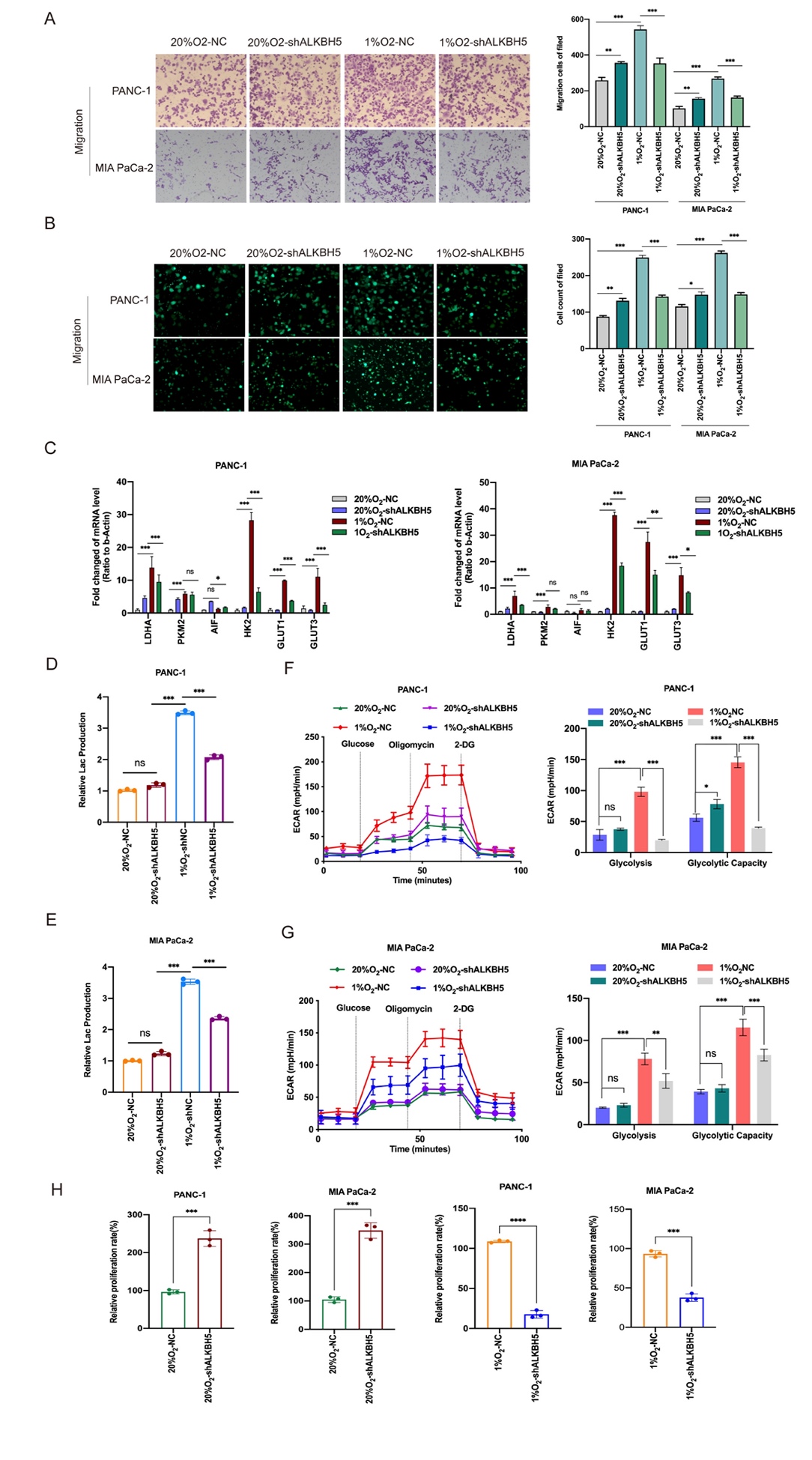


**Figure S3.** ALKBH5 regulated metastasis and glycolysis of PC cells. (**A, B**) Knockdown of ALKBH5 in PC cells exposed to either 20% or 1% O_2_ for 48h. Migration assays using Transparent PET membranes (**A**) and Fluoblock membranes (**B**) showed the migratory capability of PC cells. (**C**) PC cells with NC or ALKBH5 inhibition were incubated under 20% or 1% O_2_ for 48h. qRT-PCR was performed to determine the mRNA expression of glycolysis driver genes (LDHA, PKM2, AF, HK2, GLUT1 and GLUT3). Results were normalized to 1% O_2_-NC (mean ± SEM; n = 3). (**D, E**) Lactate production was measured in ALKBH5-knockdown PC cells incubated under normoxia and hypoxia. (**F, G**) The glycolytic metabolism of ALKBH5-knockdown PC cells was measured by Seahorse XF24 system. Glycolytic variations (**right**) of glycolysis and glycolytic capacity were summarized from raw data. The metabolic inhibitors were injected sequentially at different time points as indicated. n = 3-4 for each treatment group. (**H**) PC cells with NC or ALKBH5 overexpression were incubated under normoxia and hypoxia for 48h. Proliferative activity of PANC-1 and MIA PaCa-2 cells was assessed by CCK8 assay. n = 3 biological replicates. *P < 0.05, **P < 0.01, ***P < 0.001 and ns not significant.


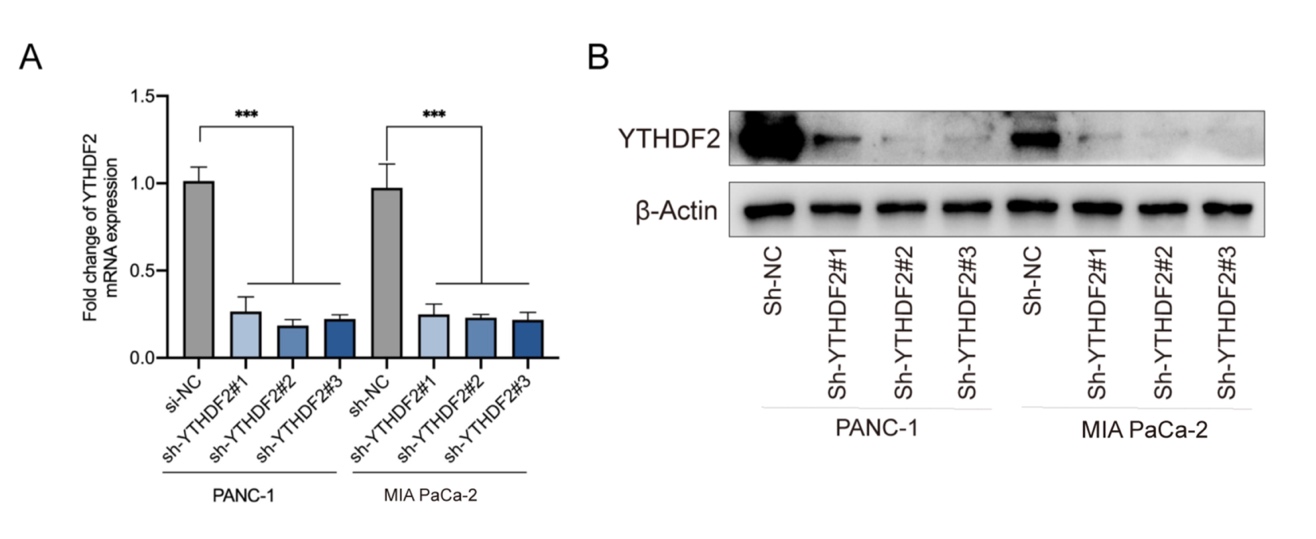


**Figure S4.** Validation of the expression level of ALKBH5 and YTHDF2 in PC cells. (**A**) qRT-PCR analysis of or YTHDF2 mRNA level in PANC-1 and MIA PaCa-2 cells. (**B**) Western blot assays were used to verify the reduced expression of YTHDF2 in PANC-1 and MIA PaCa-2 cells. *P < 0.05, **P < 0.01, ***P < 0.001 and ns not significant.


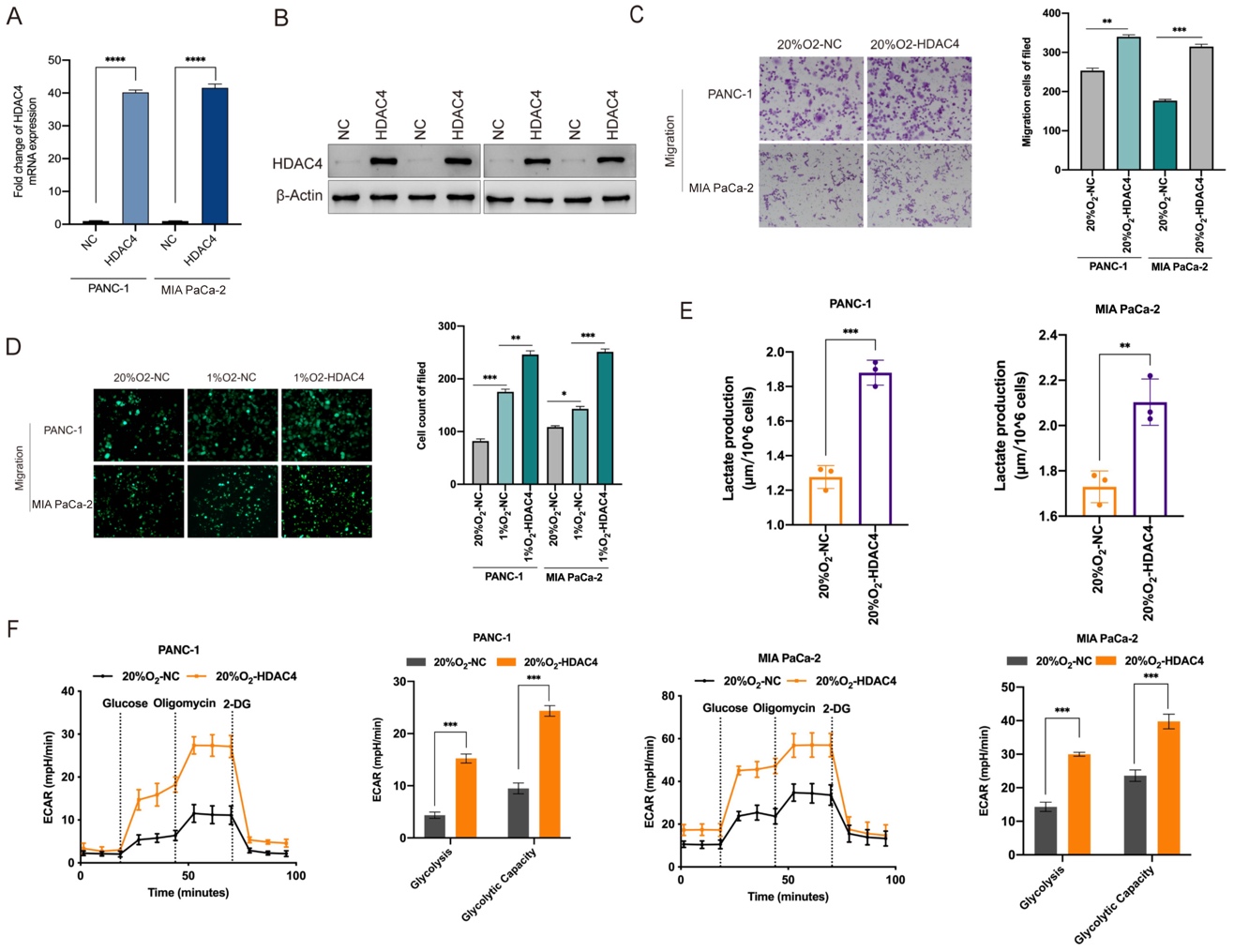


**Figure S5.** The effect of HDAC4 overexpression on glycolytic metabolism and migration. (**A, B**) qRT-PCR and Western blot assays were used to verify the mRNA levels(**A**) and protein levels (**B**) of HDAC4. (**C, D**) The cell migration abilities were investigated by migration assays using the Transparent PET membranes (**C**) and Fluoblock mebmranes (**D**). (**E**) Lactate production was measured in HDAC4-overexpression PANC-1 or MIA PaCa-2 cells. (**F**) The ECAR was measured to determine the glycolytic metabolism of HDAC4-overexpression PANC-1 or MIA PaCa-2 cells. Glycolytic variations of glycolysis and glycolytic capacity were summarized from raw data. The metabolic inhibitors were injected sequentially at different time points as indicated. n = 3-4 for each treatment group. *P < 0.05, **P < 0.01, ***P < 0.001 and ns not significant.

**
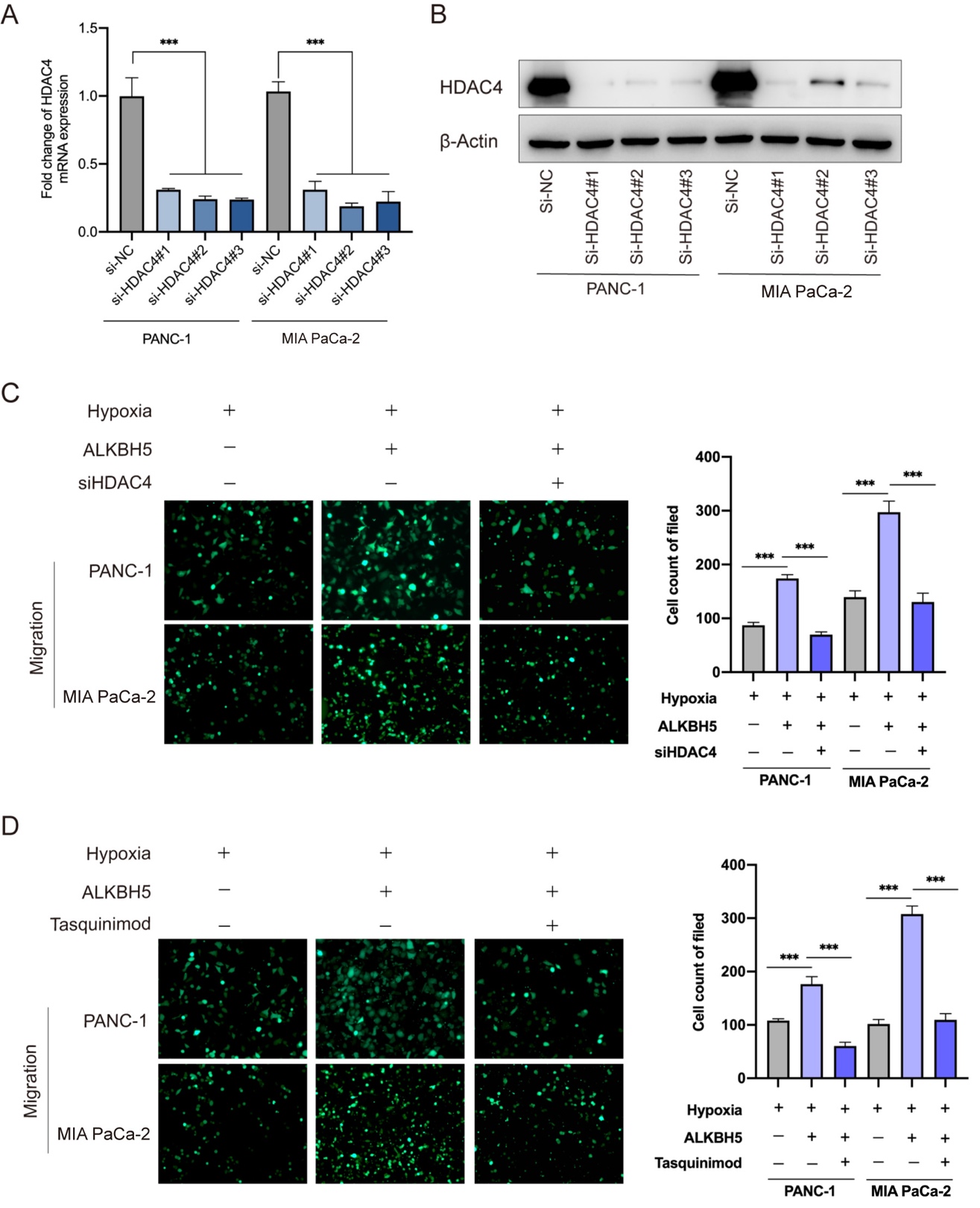
**

**Figure S6.** The effect of HDAC4 interference on migration. (**A, B**) The HDAC4 interference efficiency was verified at the mRNA levels (**A**) and protein levels (**B**) in PC cells by qRT-PCR and western blot assay, respectively. (**C, D**) The cell migration abilities were investigated by migration assays using Fluoblock membranes. *P < 0.05, **P < 0.01, ***P < 0.001 and ns not significant.

**Supplementary** **Table**

**Table S1. Primers information of qPCR experiments**

| Gene names | Sense (5’-3’) | Anti-sense (5’-3’) |
| --- | --- | --- |
| H-METTL3 | TCTGGGGGTATGAACGGGTA | CCTGTCCGAATGATGCGTTG |
| H-METTL14 | GGCAGAAGTTACGGCGACAG | ATTTAACACGGCACCAATGCT |
| H-WTAP | GCAACAACAGCAGGAGTCTG | TCGCTGGGTCTACCATTGTT |
| H-VIRMA | CACGACACAGATGCTGGACT | TCGTGCCATCGTTGGGAAAG |
| H-RBM15B | ACCTGGACCACAGCGTATCT | ATGGGGTTGCGACCAATCAC |
| H-ALKBH5 | GCCGTCATCAACGACTACCA | CGACACCCGAATAGGCTTGA |
| H-FTO | CTGCTCACTCCGGTATCTCG | GACCGTAAAGAGCCTGGTGT |
| H-LDHA | TTGTTGGGGTTGGTGCTGTTG | AAGAGCAAGTTCATCTGCCAAG |
| H-PKM2 | AATCACGCTGGATAACGCCT | TCGGCACCTTTCTGCTTCAC |
| H-AIFM1 | ATCCGTTGGAGTCAGCAGTG | ACCCAGATGTTAGAGCGTGC |
| H-HK2 | GCCCGCCAGAAGACATTAGA | GCTCAGACCTCGCTCCATTT |
| H-GLUT1 | GAGCAGCTACCCTGGATGTC | GGAAGCACATGCCCACAATG |
| H-GLUT3 | CCGCTGCTACTGGGTTTTAC | ACCGCTGGAGGATCTGCTTA |
| H-ICAM1 | AGGATGGCACTTTCCCACTG | GGAGAGCACATTCACGGTCA |
| H-CITED2 | GCGGCCAGGTTTAACAACTC | TGCTGGTTTGTCCCGTTCAT |
| H-HDAC4 | TCACTCCCTACCTGAGCACC | GGCCTGAAAGATACCAGTCTGT |
| H-HDAC7 | TCCACTGCTCCGAAAGGAGA | CTCGCTGTCATTGGGGGAG |
| H-DEPP1 | CCTCTCCTAGCCTGGATGACT | ACTGAAACGTGCGGTGATGTC |
| H-KLF4 | AATTCGCTGACCCATCCTCC | TGATGTCCGCCAGGTTGAAG |
| H-NYAP1 | CCACCATTGACGGCAACATC | GTAGCCTTGAGACCCCATCG |
| H-GAPDH | AAGAAGGTGGTGAAGCAGG | AGGTGGAGGAGTGGGTGTCG |
| H-β-actin | CACCATTGGCAATGAGCGGTTC | AGGTCTTTGCGGATGTCCACGT |

| Gene names | Sense (5’-3’) |
| --- | --- |
| shALKBH5#1 | GTCCTTCTTTAGCGACTCTTTCAAGAGAAGAGTCGCTAAAGAAGGACTTTTTT |
| shALKBH5#2 | CCTCAGGAAGACAAGATTAGATTCAAGAGATCTAATCTTGTCTTCCTGAGGTTTTTT |
| shALKBH5#3 | CCACCCAGCTATGCTTCAGATTTCAAGAGAATCTGAAGCATAGCTGGGTGGTTTTTT |
| shYTHDF2#1 | GAACGTCAAGGTCGTGGGAAA |
| shYTHDF2#2 | ACACATTCGCCTAGAGAACAA |
| shYTHDF2#3 | CCACAGGCAAGGCCCAATAAT |
| siHDAC4#1 | CCAGGCUAAAGCAGAAAGUTT ACUUUCUGCUUUAGCCUGGTT |
| siHDAC4#2 | CCAGCACAGAAGUGAAGAUTT AUCUUCACUUCUGUGCUGGTT |
| siHDAC4#3 | GUGGGUUUCAACGUCAACATT UGUUGACGUUGAAACCCACTT |
| siHIF1α#1 | GUUGCCACUUCCACAUAAUTT AUUAUGUGGAAGUGGCAACTT |
| siHIF1α#2 | CCGUAUGGAAGACAUUAAATTUUUAAUGUCUUCCAUACGGTT |
| siHIF1α#3 | CAGGCCACAUUCACGUAUATTUAUACGUGAAUGUGGCCUGTT |

**Table S2. shRNA and siRNA sequence information**
